# Krüppel-like factor 9 (KLF9) links hormone dysregulation and circadian disruption to breast cancer pathogenesis

**DOI:** 10.1186/s12935-023-02874-1

**Published:** 2023-02-23

**Authors:** Weand S. Ybañez, Pia D. Bagamasbad

**Affiliations:** National Institute of Molecular Biology and Biotechnology, University of the Philippines Diliman, Quezon City, Metro Manila 1101 Philippines

**Keywords:** KLF9, Circadian disruption, Glucocorticoid, Estrogen, Breast cancer

## Abstract

**Background:**

Circadian disruption is an emerging driver of breast cancer (BCa), with epidemiological studies linking shift work and chronic jet lag to increased BCa risk. Indeed, several clock genes participate in the gating of mitotic entry, regulation of DNA damage response, and epithelial-to-mesenchymal transition, thus impacting BCa etiology. Dysregulated estrogen (17β-estradiol, E2) and glucocorticoid (GC) signaling prevalent in BCa may further contribute to clock desynchrony by directly regulating the expression and cycling dynamics of genes comprising the local breast oscillator. In this study, we investigated the tumor suppressor gene, Krüppel-like factor 9 (*KLF9*), as an important point of crosstalk between hormone signaling and the circadian molecular network, and further examine its functional role in BCa.

**Methods:**

Through meta-analysis of publicly available RNA- and ChIP-sequencing datasets from BCa tumor samples and cell lines, and gene expression analysis by RT-qPCR and enhancer- reporter assays, we elucidated the molecular mechanism behind the clock and hormone regulation of *KLF9*. Lentiviral knockdown and overexpression of *KLF9* in three distinct breast epithelial cell lines (MCF10A, MCF7 and MDA-MB-231) was generated to demonstrate the role of KLF9 in orthogonal assays on breast epithelial survival, proliferation, apoptosis, and migration.

**Results:**

We determined that *KLF9* is a direct GC receptor target in mammary epithelial cells, and that induction is likely mediated through coordinate transcriptional activation from multiple GC-responsive enhancers in the *KLF9* locus. More interestingly, rhythmic expression of *KLF9* in MCF10A cells was abolished in the highly aggressive MDA-MB-231 line. In turn, forced expression of KLF9 altered the baseline and GC/E2-responsive expression of several clock genes, indicating that KLF9 may function as a regulator of the core clock machinery. Characterization of the role of KLF9 using complementary cancer hallmark assays in the context of the hormone-circadian axis revealed that KLF9 plays a tumor-suppressive role in BCa regardless of molecular subtype. KLF9 potentiated the anti-tumorigenic effects of GC in E2 receptor + luminal MCF7 cells, while it restrained GC-enhanced oncogenicity in triple-negative MCF10A and MDA-MB-231 cells.

**Conclusions:**

Taken together, our findings support that dysregulation of *KLF9* expression and oscillation in BCa impinges on circadian network dynamics, thus ultimately affecting the BCa oncogenic landscape.

**Supplementary Information:**

The online version contains supplementary material available at 10.1186/s12935-023-02874-1.

## Background

The circadian rhythm maintains vital homeostasis during the 24-h sleep wake cycle [1, 2]. Cell-autonomous circadian clocks are based on the molecular logic of interlocking transcriptional-translational feedback loops (TTFLs): the master regulators circadian locomotor output cycles kaput (CLOCK) and brain and muscle ARNTL-like 1 (BMAL1; also known as ARNTL) drive the expression of their negative regulators period (PER), cryptochrome (CRY), nuclear receptor subfamily 1 group D1/2 (NR1D1/2, also known as REV-ERB), and basic-loop-helix loop family members E40/41 (also known as DEC1/2), and the positive regulator retinoic acid receptor-related orphan receptor. Ultimately, this results in the rhythmic expression of clock-controlled genes and downstream cellular function in a variety of cell types, allowing circadian clocks to influence organismal physiology [3]. As such, circadian dysfunction may contribute to the development of a multitude of diseases, ranging from obesity to cancer [4].

Inherent genetic causes account for less than 10% of the etiology of breast cancer (BCa). Non-hereditary factors have been strongly linked to BCa occurrence, including exogeneous hormone intake, alcohol consumption, and altered light/dark cycles [5, 6]. Indeed, epidemiological studies and pre-clinical animal models have established the connection between altered circadian rhythms and increased BCa pathogenesis, leading to the classification of chronodisruption as a potential carcinogen by the World Health Organization [7]. Clock desynchrony may induce breast carcinogenesis through the dysregulation of different cancer regulatory pathways, including checkpoint bypass towards uncontrolled cell proliferation, genomic instability owing to impaired DNA damage response, and metastatic outgrowth [8].

Notably, maladaptive alterations to hormone signaling in BCa may contribute to further circadian dysregulation and aggravated disease progression [9]. For example, estrogen (17β-estradiol, E2) receptor (ER) signaling, which is hyperactive in most BCa cases, can induce the expression of the core clock genes *CLOCK* and *PER2* [10, 11]. Other players in the hormone-circadian crosstalk in BCa pathogenesis are glucocorticoids (GCs) that act via the GC receptor (GR). Glucocorticoid signaling in the context of BCa is paradoxical in that GR functions as an anti-tumorigenic factor in ER-dependent BCa, while it promotes metastases and therapy resistance in ER-negative BCa subtypes [12, 13].

At the intersection of circadian regulation and hormone signaling is the Krüppel-like factor 9 (*KLF9*) gene, which encodes for a zinc finger transcription factor (TF) that is regulated in a circadian manner by the CLOCK/BMAL1 complex in the hippocampus, epidermis, and liver [14–16]. The gene is directly induced by GR signaling in different cellular contexts [17, 18], and KLF9 antagonizes ER-mediated transcriptional activity [19]. In BCa, KLF9 predominantly acts as a transcriptional repressor that targets genes involved in proliferation and metastases, most notably cyclin D1 and matrix metalloproteinase 9 [20, 21]. Despite several lines of evidence supporting the coordinate regulation of *KLF9* by hormone signaling and the circadian rhythm, and KLF9 modulation of cellular circadian clock and CLOCK/BMAL1 target genes [16], the exact role of KLF9 in the crosstalk and coincident dysregulation between the two signaling axes in BCa has yet to be explored.

In this study, we provide evidence for a role of KLF9 in the hormone-circadian axis in BCa development. We sought to (1) decipher the regulatory logic of GC- and CLOCK/BMAL1-dependent expression of *KLF9* in mammary epithelial cells, (2) characterize its reciprocal impact on the core clock machinery, and (3) investigate its role in BCa progression. We determined that *KLF9* expression in breast epithelial cell lines is induced by GR through multiple GC-regulated enhancers and is refractory to regulation by liganded ER. *KLF9* mRNA is expressed in a circadian fashion in non-malignant MCF10A cells, likely mediated through direct transcriptional activation by CLOCK/BMAL1 through an upstream enhancer. However, this rhythmic oscillation of *KLF9* mRNA is abolished in the highly aggressive MDA-MB-231 line. In turn, KLF9 may influence the baseline expression of the genetic components of the cellular circadian oscillator and their response to GC- and E2-induced transcriptional changes in BCa. Lastly, we established that KLF9 suppresses the oncogenic progression in BCa regardless of molecular subtype. In luminal ER + MCF7 cells, KLF9 cooperated with GC to effect tumor suppression, whereas KLF9 restrained the GC-induced oncogenic effects in triple-negative MCF10A and MDA-MB-231 cells.

## Materials and methods

### Cell culture

Three human mammary epithelial cell lines were used in this study, namely, MCF10A (RRID:CVCL_0598) [22], MCF7 (RRID:CVCL_0031) [23], and MDA-MB-231 (RRID:CVCL_0062) [24]. MCF10A cells represent non-malignant breast epithelial cells of basal origin that express GR but not ER, progesterone receptor (PR), or human epidermal growth factor receptor 2 (HER2) (triple-negative) [25]. Cellular responses, especially to GCs, modeled in MCF10A have been shown to reflect the physiology in normal mammary epithelia [26, 27]. MCF7, on the other hand, is a poorly invasive BCa line that belongs to the luminal A molecular subtype in account for its endogenous expression of ER and PR [28]. Growth of MCF7 cells is dependent on E2 in culture, making them the conventional model for investigating E2 response and the effects of E2 blockade in BCa [29, 30]. Finally, MDA-MB-231 is a triple-negative BCa (TNBC) line used to model late-stage, highly aggressive BCa [31]. The cell line is enriched for markers associated with epithelial-to-mesenchymal transition and also exhibits gene expression signatures associated with BCa stem cells [32]. Cell lines were authenticated by Macrogen (Korea) using short tandem repeat profiling (Powerplex 21 System, Promega) and tested negative for mycoplasma contamination using the Microsart AMP Mycoplasma Kit (Sartorius).

MCF10A cells and MDA-MB-231 cells were cultured as previously described [33]. MCF7 cells were cultured in MEM with 10% fetal bovine serum (FBS; Gibco, 10270106) and 10 μg/mL insulin, Human Recombinant Zinc Solution (Gibco, 12585-014), and 1X penicillin–streptomycin. All cell lines were incubated in a humidified environment at 37 ℃ and 5% CO_2_.

### Plasmid constructs

#### Enhancer reporter constructs

Three putative enhancer regions in the *KLF9* locus were cloned into the pGL4.23[*luc2*/minP] luciferase vector (Promega, E8411). The 693 bp extended KLF9 synergy module (eKSM) [17] is located 4.3 kb upstream of the *KLF9* transcription start site (TSS). Upstream of the eKSM is the adjacent KSM (aKSM), a 424 bp fragment located 5.2 kb upstream of the TSS. Lastly, the KLF9 distal enhancer (KDE) is a 542 bp fragment located 65.98 kb upstream of the TSS. The candidate *cis-*regulatory elements were amplified from MCF10A genomic DNA through polymerase chain reaction (PCR) using Platinum™ SuperFi™ PCR Master Mix (Thermofisher, 12358010) with oligonucleotide primers in Additional file 1: Table S1. The DNA fragments were then digested and ligated into *Kpn*I and *Hind*III sites upstream of the *luc2* gene of the pGL4.23[*luc2*/minP] vector.

#### *CLOCK* and *BMAL1* expression constructs

DNase-treated MCF10A RNA was used as template to generate the cDNAs corresponding to the open reading frames (ORFs) of *CLOCK* (NM_004898.4 → NP_001254772.1) and *BMAL1* (isoform c; NM_001297719.2 → NP_001284648.1) (Additional file 1: Table S1). After two rounds of PCR (Ext, Int primers), the *BMAL1* ORF was then subcloned into the pCMV6-entry construct (Origene, PS100001) for transient overexpression while the *CLOCK* ORF was subcloned into the pCMV6-entry and pLenti-c-Myc-DDK vector (Origene, PS100092) using the *Asi*SI and *Mlu*I sites for stable overexpression in MCF10A cells.

#### Lentiviral *KLF9* shRNA and expression constructs

A vector bearing shRNA targeting *KLF9* was generated [shKLF9-3: TRCN0000013630, Genetic Perturbation Platform shRNA library, Broad Institute] by ligating annealed shRNA oligonucleotides (Additional file 1: Table S1) into the pLKO.1 vector (RRID:Addgene_8453) at the *Age*I and *Eco*RI sites. The pLKO.1-scrambled shRNA (RRID:Addgene_1864) was used as a negative control.

The human KLF9 protein was also stably overexpressed in the three breast epithelial lines. To this end, the *KLF9* ORF (NM_001206.4 → NP_001197.1) was cloned into the *Asi*SI and *Mlu*I sites of the pLenti-c-Myc-DDK vector (Origene, PS100092) in a similar method to the generation of CLOCK/BMAL1 expression vectors (Additional file 1: Table S1). The empty pLenti vector served as a negative control.

### In silico analysis of *KLF9* expression in breast tumor samples

Publicly available gene expression datasets were analyzed to evaluate *KLF9* expression across tumor samples and normal breast tissues. Genotype-Tissue Expression Project (GTEx) [34] data for normal breast tissue and breast invasive carcinoma RNA-seq data from the Cancer Genome Atlas Research Network (TCGA, http://www.cancer.gov/tcga) [35] were accessed through the Gene Expression Profiling Interactive Analysis (GEPIA) tool [36]. The Breast Cancer Gene-Expression Miner 4.5 expression module (bcGenExMiner v4.7 database) [37] was used to evaluate *KLF9* expression across BCa molecular subtypes (normal-like, luminal A, luminal B, HER2-overexpressing, and basal-like) from TCGA and Sweden Cancerome Analysis Network – Breast (SCAN-B) RNA-seq data [38]. We also assessed alterations in *KLF9* expression upon 10 nM E2 treatment by analyzing RNA-seq data from Baran-gale et al. [39], plotted as transcript per million reads (TPM) over a 24 h time-course.

### Hormone treatment and gene expression analysis

#### Hormone-dependent expression

Dose–response changes in gene expression subsequent to hydrocortisone (CORT; Sigma H0888) treatment was evaluated in the three human mammary lines. MCF10A, MCF7, and MDA-MB-231 cells were seeded in 12-well plates at a density of 2.5 × 10^5^ cells/well in their respective complete media. Upon reaching ~ 70% confluency, the cells were starved overnight in serum-free basal media and treated with increasing concentrations of CORT for 2 h as previously described [33].

To determine if *KLF9* is a direct target of GR, MCF10A cells were first seeded into 12-well plates at a density of 2.0 × 10^5^ cells per well in complete media. The cells were then starved overnight, pre-treated with the protein synthesis inhibitor cycloheximide (CHX; Sigma 01,810) prior to CORT treatment as previously described [33]. To determine if CORT-dependent induction of *KLF9* is mediated specifically by the GR, MCF10A cells were seeded at the same density as in the CHX experiment, starved overnight and pre-incubated with the GR-specific antagonist mifepristone (MIF; Sigma, M8046) prior to treatment as previously described [33].

Dose–response analysis of gene expression subsequent to E2 (Sigma, E8875) treatment was also performed in ER-expressing MCF7 cells. The cells were seeded in 12-well plates at a density of 2.5 × 10^5^ cells/well. E2 was dissolved in ethanol and basal media at various concentrations (3, 10, 30, 100, 300, and 1000 nM; final ethanol concentration = 0.0026%). Cells were incubated with the mixture for 2 h prior to harvest for RNA extraction. Each treatment was done in quadruplicate and the experiment was performed twice.

To investigate KLF9-induced alterations in the hormone response of GC/E2-regulated clock genes, we also treated mammary epithelial cells stably overexpressing KLF9 with CORT or E2. For GC response, we treated *KLF9-*expression-vector transduced MCF10A and MDA-MB-231 lines with 100 nM CORT for 2 h prior to gene expression analysis. We excluded MCF7 from this analysis since it expresses low GR mRNA compared to the other cell lines. For E2 response, we treated ER + MCF7 KLF9-overexpressing cells with 1 μM E2 for 24 h prior to gene expression analysis.

Total RNA was extracted from cell lines using the TRIzol reagent (Invitrogen, 15596-018) following the manufacturer’s protocol. Synthesis of cDNA and gene expression analysis were performed as described previously [33]. Primers used to quantify mRNA levels were designed to span an exon-exon boundary while primers used to quantify pre-mRNA were designed to span intron and exon sequences (Additional file 1: Table S2). For measurement of enhancer RNA (eRNA) levels, we designed oligonucleotide primers to amplify 80–110 bp fragments from the enhancer sequences. Minus reverse transcriptase controls were included to account for possible genomic DNA contamination.

#### Time-course analysis of circadian gene expression

MCF10A and MDA-MB-231 cells were synchronized based on an entrainment protocol developed by Balsalobre and colleagues [40]. Cells were seeded at 5.0 × 10^5^ cells per well in complete media. Upon reaching ~ 90–100% confluency, cells were starved overnight in serum-free basal media and synchronized by treatment with 1 μM CORT for 2 h as previously described [16, 40]. After the CORT pulse, the cells were washed with 1X DPBS and the entrainment medium was then replaced with serum-free medium. Timepoints were taken directly after the pulse (t = 0 h) and every 4 or 6 h thereafter for 48 h by harvesting the cells for gene expression analysis.

To analyze circadian gene expression subsequent to entrainment of mammary epithelial cells, we applied the previously described methods [16, 41] where a nonlinear fit on linearly detrended data was performed using the equation below in GraphPad Prism version 8.0 (GraphPad Software, La Jolla, CA, US, www.graphpad.com). Initial values were defined as the following: baseline = minimum gene expression value in the entire dataset, amplitude = difference in minimum and maximum values, and phase shift = time value at maximum gene expression.

$$Y=Baseline+Amplitude*\mathrm{cos}(Frequency\cdot X+Phase shift)$$

Curve-fitting was performed on abridged data (t = 12 h to t = 44 h) to improve reliability of regression analyses as preliminary cosine-wave regression on the complete time-series datasets of both *BMAL1* and *KLF9* failed to predict oscillation based on the initial parameters defined. The CircWave software (https://clocktool.org/index.php/clock-modules/clock-tools/item/circ-wave.html) [42] was used to validate rhythmicity of clock gene expression (limited to 24-h period, t = 12 h to t = 36 h). CircWave fits a fundamental sinusoidal wave through individual gene expression data in each timepoint, and subsequently compares this with a horizontal line through the dataset mean. If the harmonic regression is significantly different from the horizontal line, then the gene is considered rhythmic. CircWave provides the *p-*value and *r*^*2*^ of the curve fit, as well as the peak phase (center of gravity). Amplitudes of each CircWave curve-fit was calculated as percentage of the data mean (see equation below) based on previous literature [43, 44] (Additional file 1: Table S3).

$$Amplitude=\frac{MAX-MIN}{Mean}*100\%$$

### Identification and validation of *KLF9* enhancer regions

To investigate putative *cis-*regulatory elements in the *KLF9* locus that respond to both GC and circadian regulation, we analyzed GR genome association through publicly available datasets [GR ChIP-seq; MCF10A: GSE102355 [45], MDA-MB-231: GSE56022 [46]], along with CLOCK, ERα, and KLF9 ChIP-seq on MCF7 [CLOCK: GSE127640, [47]; ERα: GSE135340 [48]; KLF9: (GSE105301) [47]]. We also evaluated the presence of active chromatin marks [47] and long-range interactions [49] and mapped these to the human February 2009 (GRCh37/hg19) Assembly using the UCSC Genome Browser [50]. The presence of GC and E2 response elements (GREs and EREs, respectively; Table 1) in each candidate region was then evaluated through LASAGNA 2.0 motif binding search [51]. CLOCK-binding sites were manually determined based on previously derived sequences: EboxA = CACGTG, EboxB = CACGTT or AACGTG, EboxC = CACGCG, EboxE = CACGAG in order of decreasing induction by CLOCK in a reporter enhancer assay as previously described [15].

**Table 1:** LASAGNA motif binding search of GREs and EREs in the candidate enhancer regions

Name of TF model	Position	Sequence	Strand	Score	*P*-value	*E-*value
GRα (T00337)	eKSM (−4602 bp)	ggagcttgatgttcc	−	152.1	2.5 × 10^–5^	0.0165
	aKSM (−5467 bp)	ggaacagtttgtcct	+	137.82	0.0004	0.157
	KDE GRE1 (−66,198 bp)	atcaacagcatgatct	−	146.27	0.000125	0.064
	KDE GRE2 (−66,248 bp)	cagtacagaatgttct	+	224.4	0	0
ER (M00191)	eKSM ERE (−4696 bp)	ccacgcccacgtgagctaa	+	8.19	0.00095	0.63

Positions are indicated are either in the ± strand and are relative to the TSS which is set as the zero position. Scores are obtained for the GR-alpha transcription factor model from the TRANSFAC database (T00337) or ER (M00191) based on position-specific scoring matrices. The *P*-value is empirically determined from the position-specific scoring matrices scores of individual nucleotides in the sequence relative to the TF model and is indicative of the probability of observing a score equal to or higher than the score by chance. The E-value considers the length of the genomic region being tested and gives the number of expected times a hit of the same or higher score is found in the genomic region by chance [51]

For the dual luciferase assays, MCF10A, MCF7 and MDA-MB-231 cells were seeded in 24-well plates at a density of 1.0 × 10^5^ cells/well. Upon reaching 70% confluency, the cells were then transfected, and media was changed into DMEM/F12 with 5% charcoal stripped (CS)-horse serum (HS) for MCF10A and MEM/RPMI-1640 with 10% CS-FBS for MCF7 and MDA-MB-231 cells. Cells were then transfected in quadruplicates, with 475 ng pGL4.23-eKSM/aKSM/KDE or the positive control pGL4.23-ERRFI1 downstream enhancer (EDE) [33], and 25 ng pRL-TK (Promega, E2241) as a normalization control using the XtremeGENE HP DNA Transfection Reagent (Roche, 6366236001) as per the manufacturer’s protocol. Following overnight incubation with the transfection complex, the cells were treated with 1 μM MIF and 300 nM (MCF10A, MDA-MB-231) or 1 μM (MCF7) CORT as described previously [33]. Luciferase activity was measured using the Dual-Luciferase Reporter Assay System (Promega, E1980) and the Fluoroskan™ FL Microplate Luminometer (ThermoScientific).

### Lentiviral-mediated knockdown of *KLF9* and forced expression of *KLF9 *and *CLOCK*

Lentiviral particles were packaged in HEK293T cells by transfecting the pLKO.1 or pLenti constructs with viral packaging plasmids (pHCMVG, pRSV-rev, pMDLg/RRE), filtered, and harvested as described previously [33]. MCF10A, MCF7, and MDA-MB-231 cells were transduced by incubating them with viral soup with 8 μg/mL polybrene (Merck, TR-1003-G) for 24 h and transduced cells were selected using complete media with 2.0 μg/mL puromycin (selection media; Gibco, A11138-03). Knockdown or overexpression of *KLF9* was validated through RT-qPCR. This was further confirmed by measuring the mRNA expression of KLF9-repressed target genes in *KLF9* knockdown or overexpression cell lines.

### *BMAL1* and *CLOCK* transfection

MCF10A and MDA-MB-231 cells stably overexpressing *CLOCK* were seeded in 6-well plates at a density of 3.0 × 10^5^ in complete media. At 70% confluency, cells were transfected with 1.5 μg of pCMV6-*BMAL1* or pCMV6-entry vector and transfection complexes were prepared according to the XtremeGENE HP (Roche) DNA transfection protocol at a 1:2 DNA to transfection reagent ratio. Following 24 h incubation, cells were harvested with TRIzol for RNA extraction.

For the dual luciferase assay, MCF10A cells were seeded in 24-well plates at a density of 1.0 × 10^5^ cells/well. The cells were then transfected in quadruplicates using the XtremeGENE HP DNA Transfection Reagent, with either 300 ng pCMV6-entry or 150 ng each of pCMV6-BMAL1/CLOCK, along with 190 ng pGL4.23-eKSM, which contains a canonical E-box and three other non-canonical E-boxes, and 10 ng pRL-TK, which served as a normalization control. Following overnight incubation with the transfection complex, luciferase activity was measured using the Dual-Luciferase Reporter Assay System and the Fluoroskan™ FL Microplate Luminometer as previously described.

### Colony formation assay

MCF10A (3.0 × 10^3^ cells/well), MCF7 (6.0 × 10^2^ cells/well), and MDA-MB-231 (1.5 × 10^3^ cells/well) knockdown or overexpression lines were seeded into 6-well plates in complete media. The cells were starved in basal media supplemented with 2.5% CS-HS (MCF10A) or 2.5% CS-FBS (MCF7 and MDA-MB-231) overnight and treated with 100 nM CORT. Hormone was replenished every third day and cells were fixed, stained, and analyzed as described previously [33]. Colonies (50 cells/colony) were manually counted using Vision SX45 Stereomicroscope. Experiments were performed in quintuplicate and were repeated at least twice.

### Cell viability assay

MCF10A (3.0 × 10^3^ cells/well), MCF7 (2.0 × 10^3^ cells/well), and MDA-MB-231 (2.0 × 10^3^ cells/well) knockdown or overexpression lines were seeded into 96-well clear-bottom black plates (Corning, 3603) in complete media. The cells were then starved in basal media supplemented with 5% CS-HS (MCF10A) or 10% CS-FBS (MCF7 and MDA-MB-231) overnight. The following day, baseline cell viability (t = 0 h) was measured after incubation with PrestoBlue reagent (Invitrogen, A13262) and cells were then treated with vehicle or CORT (100 nM; 0.0036% final ethanol concentration). Hormone was replenished and cell viability was measured until the 96 h timepoint as described previously [33]. Each treatment had 5 replicates for each timepoint, and all experiments were at least repeated twice.

### CellEvent caspase-3/7 apoptosis assay

MCF7 and MDA-MB-231 knockdown lines were seeded into 96-well clear-bottom black plates (Corning, 3603) at 8.0 × 10^3^ cells/well in complete media. At 90% confluency, the cells were starved in 10% CS-FBS overnight and treated with CORT (100 nM) and doxorubicin (DOX; 1 μM for MCF7 and 10 μM for MDA-MB-231; Sigma, D1515). Apoptosis was measured 48 h post-treatment by labeling cells with 5 μM CellEvent Caspase-3/7 Detection Reagent (Invitrogen, C10423) as described previously [33]. Each treatment was performed with 5 replicates and experiments were performed at least twice.

### Wound healing assay

MCF7 and MDA-MB-231 knockdown lines were seeded at 2.5 × 10^5^ cells/well. When the cells have reached confluency, they were starved in basal media with 1% (MCF7) or 0.5% (MDA-MB-231) CS-FBS, pre-incubated with 10 μg/mL mitomycin C (Millipore, 475820) for 2 h prior to creating a scratch wound using a P200 tip. The cells were then treated with 500 nM CORT and wound closure was monitored as described previously [33].

### Statistical analysis

Significant differences across BCa molecular subtypes were assessed through Welch’s test for global significant difference between groups and the Dunnett-Tukey–Kramer’s test was used to test for multiple pairwise comparisons (different letters above the means indicate significant difference; *P* < 0.05). Data from gene expression analysis (normalized either to *18S rRNA* or *GAPDH* which were unchanged by treatments), dual luciferase assays (Firefly luciferase counts divided by *Renilla* luciferase counts), and cell proliferation and viability (fluorescence signal at timepoint divided by signal at 0 h) were log_10_ transformed before statistical analysis. The dose–response, MIF, and dual luciferase assay data were analyzed using one-way analysis of variance (ANOVA) followed by Tukey’s post-hoc test (*P* < 0.05; means with the same letter are not significantly different), while data from the CHX assay were analyzed using the Student’s unpaired *t*-test (^*^P < 0.05, ^**^P < 0.01, ^***^P < 0.001, ^****^P < 0.0001). Data from the hormone treatment of *KLF9*-overexpressing lines, colony formation, cell proliferation, and cell viability assays were analyzed using two-way ANOVA to test for main effects of CORT treatment and *KLF9* expression, followed by Student’s unpaired *t*-test to determine effects of CORT within an shRNA or overexpression line (^*^P < 0.05, ^**^P < 0.01, ^***^P < 0.001, ^****^P < 0.0001) and effect of *KLF9* expression between the same hormone treatment (^#^*P* < 0.05, ^##^*P* < 0.01, ^###^*P* < 0.001, ^####^*P* < 0.0001). Apoptosis assay data were analyzed using one-way ANOVA for effects of CORT/DOX treatment within an shRNA type, followed by Student’s *t*-test for effects of knockdown within a treatment. All statistical analyses were done using GraphPad Prism version 8.0 (GraphPad Software, La Jolla, CA, US, www.graphpad.com), and *P* < 0.05 was accepted as statistically significant.

## Results

### Hormone-dependent regulation of the *KLF9* gene in the breast

**Fig. 1:**
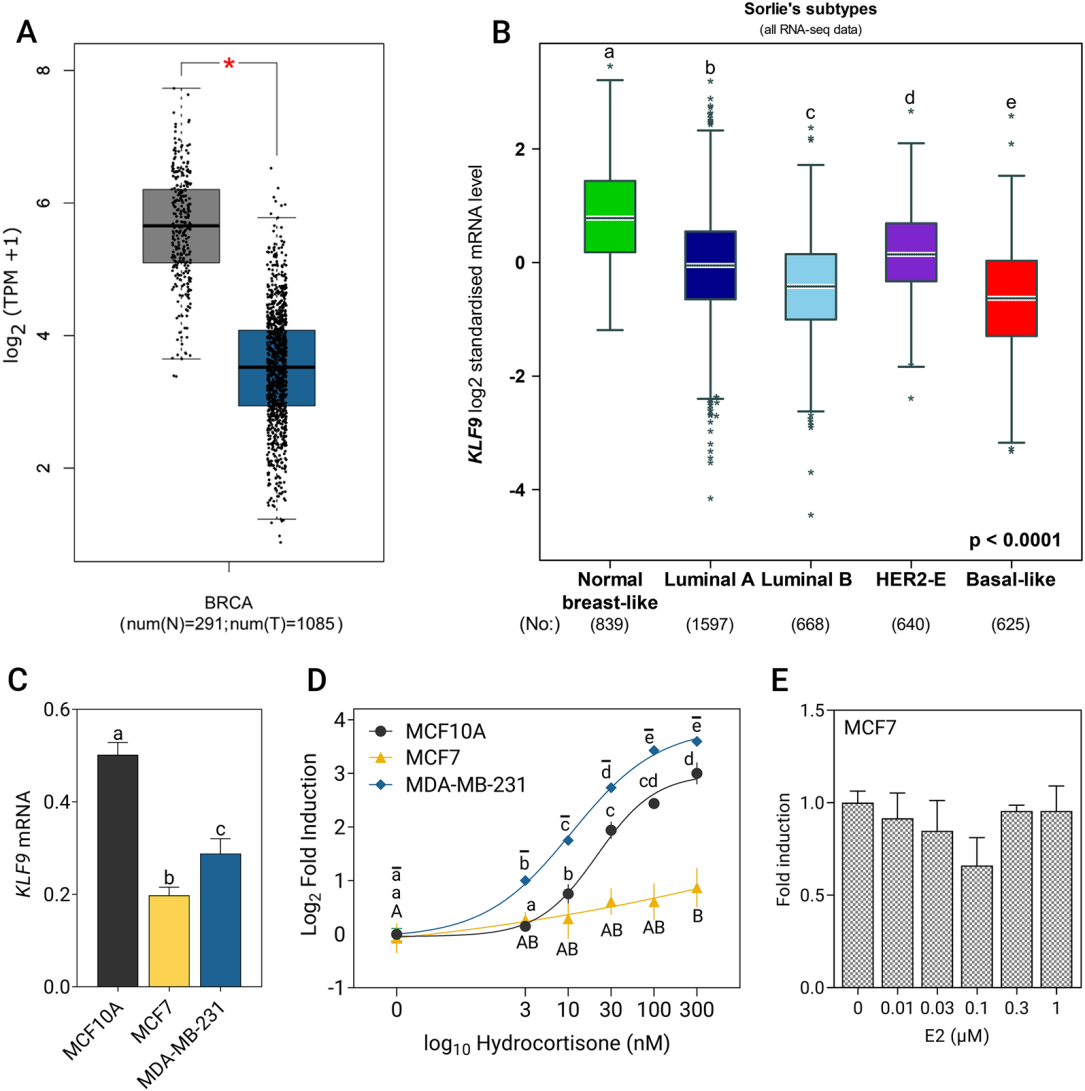
*KLF9* is downregulated in breast tumors and induced by CORT in BCa cell lines. **A** *KLF9* expression in breast cancer (BCa) samples relative to normal tissue equivalents based on analysis conducted using the GEPIA web tool [36] (TPM = transcripts per million; BRCA = breast cancer). **B** *KLF9* expression evaluated across BCa molecular subtypes assessed using the bcGenExMiner v4.7 tool [37] (Welch’s test: *P* < 0.0001, followed by Dunnett-Tukey–Kramer’s test for multiple pairwise comparisons: *P* < 0.05; boxplots with different letters are significantly different). **C** Baseline expression of *KLF9* in the three breast epithelial cell lines as measured through RT-qPCR (one-way ANOVA; *P* = 0.0004). **D** MCF10A, MCF7, and MDA-MB-231 cells were treated with increasing concentrations of CORT for 2 h. In MCF10A and MDA-MB-231, dose-dependent increase in *KLF9* transcript levels was observed upon CORT treatment. In MCF7, a significant increase in *KLF9* mRNA was observed only at 300 nM CORT (one-way ANOVA; MCF10A, MDA-MB-231: *P* < 0.0001; MCF7: *P* < 0.0405). **E** MCF7 cells were treated with increasing doses of E2 for 24 h. *KLF9* expression remained unchanged with E2 treatment across all concentrations (one-way ANOVA; *P* = 0.231). Dose–response curves in the CORT treatment were fitted by nonlinear regression and dots represent the log_2_(fold induction) ± SEM while bars represent mean ± SEM with statistical significance indicated by letters above the means [CORT treatment: MCF10A (lowercase), MCF7 (uppercase), MDA-MB-231 (overline)] in one-way ANOVA followed by Tukey’s post-hoc test: *P* < 0.05; means with the same letter are not significantly different. All experiments were performed with N ≥ 3 replicates

Based on publicly available RNA-seq datasets from the GTEx and TCGA breast invasive carcinoma, we found that *KLF9* expression is significantly downregulated in tumor samples in comparison to normal tissue controls (Fig. 1A). Stratifying tumor samples into molecular subtypes based on transcriptomic data from TCGA and SCAN-B, expression of *KLF9* was moderately downregulated in subtypes associated with increasing disease severity and worse prognosis (Fig. 1B). This pattern of expression was reflected in BCa cell lines, with the non-malignant MCF10A cells exhibiting the highest *KLF9* mRNA levels (Fig. 1C).

The *KLF9* gene is an established direct GR target in neuronal, skin, and lung cells [14, 18, 52]. To evaluate the GC-dependent regulation of *KLF9* in the breast, we treated BCa lines with increasing doses of CORT (0–300 nM) for 2 h. In MCF10A and in the highly aggressive MDA-MB-231 cells, *KLF9* mRNA expression increased with increasing CORT concentrations (Fig. 1D; MCF10A: EC_50_ = 21.71, MDA-MB-231: EC_50_ = 10.93). On the other hand, a significant induction in *KLF9* expression was only seen in MCF7 cells treated with 300 nM CORT (Fig. 1D, EC_50_ = 46.31), which may be attributed to significantly lower *NR3C1* (GR) transcript levels in MCF7 cells (Additional file 2: Fig. S1).

Analysis of transcriptome data in ER + MCF7 cells [39] revealed that 10 nM E2 treatment of MCF7 cells led to a rapid and transient increase in *KLF9* mRNA that returned to baseline levels by the first hour of treatment and steadily declined over the course of 24 h (Additional file 3: Fig. S2A). To further explore the possible E2-dependent regulation of *KLF9*, we performed a dose–response experiment on ER + MCF7 cells incubated with increasing concentrations E2 for 24 h. However, we observed that *KLF9* expression remained generally unaltered with E2 treatment (Fig. 1E) in contrast with the E2-dose dependent increase in mRNA level of the direct ER target *GREB1* (Additional file 3: Fig. S2B, C).

### *KLF9* is directly regulated by GR via GC-responsive enhancers

**Fig. 2:**
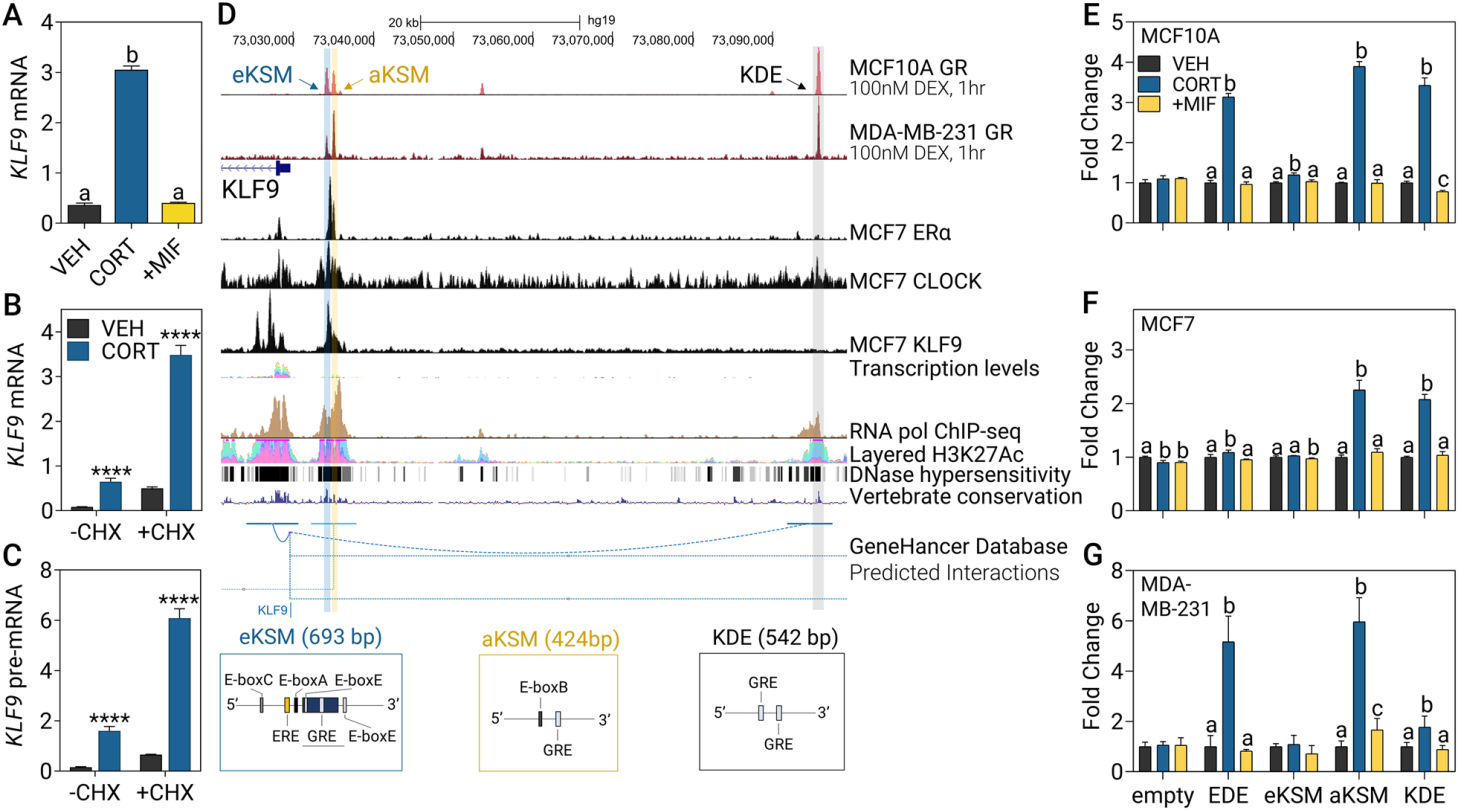
CORT-dependent induction of *KLF9* is mediated by the GR through coordinate enhancer activation. **A** Pre-treatment with 1 μM of the GR-specific antagonist MIF for 1 h before addition of 100 nM CORT for 2 h abolished CORT-dependent *KLF9* induction (one-way ANOVA; *P* < 0.0001). **B**, **C** MCF10A cells were pre-incubated with 100 μg/mL CHX for 30 min before treatment with 300 nM CORT for 2 h and *KLF9* **B** mRNA and **C** pre-mRNA expression was measured through RT-qPCR (Student’s *t-*test; *P* < 0.0001). **D** The *KLF9* locus and surrounding nongenomic regions were visualized in the UCSC genome browser [50] mapped to the GRCh37/hg19 assembly. Highlighted are eKSM, aKSM, and KDE, all located upstream of the *KLF9* transcription start site. **E**–**G** The aKSM, eKSM, and KDE were cloned into the pGL4.23 luciferase construct and transfected into **E** MCF10A, **F** MCF7, and **G** MDA-MB-231 cells. An empty pGL4.23 vector were used as a negative control while the EDE [33] served as a positive control. Cells were then treated with vehicle (VEH), CORT (300 nM: MCF10A, MDA-MB-231; 1 μM: MCF7) or CORT plus MIF (1 μM; + MIF) for 20 h to evaluate GR-specific induction of luciferase activity for each enhancer (one-way ANOVA within a construct, followed by Tukey’s post-hoc test: *P* < 0.05; means with the same letter are not significantly different). Bars represent mean ± SEM and all experiments were performed with N ≥ 3 replicates

Treatment of MCF10A with the GR-selective antagonist MIF completely abolished the CORT-induced increase in *KLF9* mRNA indicating that CORT-dependent regulation of *KLF9* is mediated by and specific to the GR, and not the mineralocorticoid receptor (Fig. 2A). In addition, treatment with the protein synthesis inhibitor CHX did not affect the CORT-dependent increase in *KLF9* mRNA (Fig. 2B) and pre-mRNA (Fig. 2C) supporting that GR directly upregulates the transcription of the *KLF9* gene in MCF10A cells.

We then sought to identify putative *cis-*regulatory elements that can mediate response to GC, E2, and circadian signaling. We performed in silico analysis of GR, CLOCK, and ER ChIP-seq data [45, 46, 48], active chromatin marks (H3K27Ac, DNase I hypersensitivity, RNA pol II binding) [45, 53], evolutionary conservation among vertebrates [53], and predicted long-range interactions with the *KLF9* promoter from the GeneHancer database [49], which altogether constitute features of candidate GC-, E2-, and circadian clock-responsive enhancer elements. Based on the GR ChIP-seq data and sequence analysis with LASAGNA 2.0 [51], there was negligible GR localization and no GRE identified in the proximal *KLF9* promoter until the region 3 kb upstream of the TSS (Additional file 4: Fig. S3). With this, we focused on three candidate regulatory elements which show preferential GR localization upon treatment with the synthetic GC analog dexamethasone (DEX) in both MCF10A and MDA-MB-231 cells, along with presence of active chromatin marks within each putative enhancer (Fig. 2D). First, the previously described mouse KSM [17], was extended to include three additional CLOCK-binding motifs (E-boxes) and an ERE half-site. Herein referred to as eKSM, it is located 4.3 kb from the TSS and harbors a canonical GRE and a GRE half-site (Table 1, Additional file 5: Fig. S4). Upstream of the eKSM located 5.2 kb from the TSS and referred to as aKSM is another candidate enhancer enriched for GR and CLOCK localization and contains a non-canonical E-box and a GRE. Finally, the KDE, a TF binding hotspot located 65.98 kb upstream of the *KLF9* TSS, is another potential *cis-*regulatory element containing two GREs.

We cloned each of the identified putative enhancer regions into a luciferase reporter construct and evaluated CORT-mediated transactivation in the three mammary epithelial cell lines. In MCF10A cells, a robust increase in luciferase activity in cells transfected with the aKSM and KDE constructs was observed upon CORT treatment and the induction was abolished with the addition of MIF (Fig. 2E). Notably, transactivation of the eKSM construct, although significant, was only at 1.19-fold relative to ~ threefold activation for the other enhancer-reporter constructs. In MCF7 cells, GR-specific transactivation of aKSM and KDE is conserved, while the eKSM was not induced by CORT (Fig. 2F). Lastly, in the MDA-MB-231 line, both aKSM and KDE exhibited CORT-dependent transactivation, although the fold increase in luciferase activity for the KDE construct was only 1.7-fold in comparison to ~ threefold induction in normal MCF10A and luminal MCF7 cells (Fig. 2G). The *ERRFI1* downstream enhancer (EDE) [33] was induced in all three cell lines and served as the CORT-responsive positive control.

Owing to the localization of RNA pol II at the aKSM, eKSM, and KDE (Fig. 2D), we also investigated whether these candidate enhancers are transcribed into non-coding RNA. In MCF10A cells, hormone response of all three eRNAs was similar to that of the *KFL9* mRNA, such that eKSM (Additional file 6: Fig. S5A, B), aKSM (Additional file 6: Fig. S5C, D), and KDE (Additional file 6: Fig. S5E, F) eRNA expression was induced by CORT in a GR-specific manner and was resistant to protein synthesis inhibition.

### Circadian expression of *KLF9* in mammary epithelial cells

**Fig. 3:**
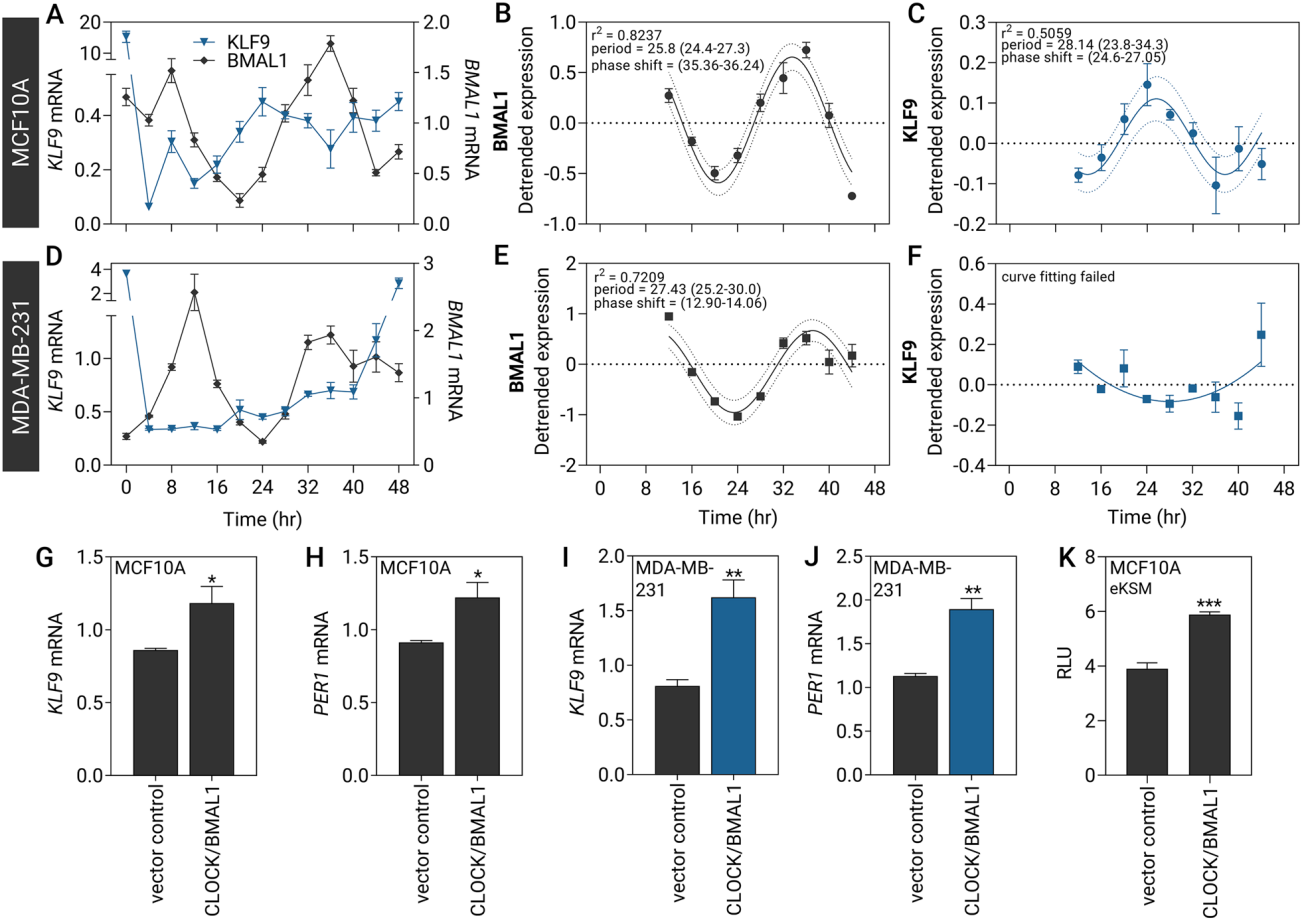
Rhythmic expression of *KLF9* in normal MCF10A cells is abolished in MDA-MB-231 TNBC line. **A**–**C** MCF10A and **D**–**F** MDA-MB-231 cells were pulsed with 1 μM CORT for 2 h to synchronize circadian gene expression prior to collection of RNA every 4 h. **A** Circadian expression of *KLF9* (blue) was antiphase with the expression of *BMAL1* (ARNTL, black), as assayed through RT-qPCR. **B**,** C** A cosine-wave curve was fitted to the expression data of **B** *BMAL1* and **C** *KLF9* to determine the period, phase shift, and goodness-of-fit r^2^ of the oscillation. **D** No apparent phasic relationship exists between *BMAL1* and *KLF9* expression in MDA-MB-231 cells. **E** Cosine-wave regression determined oscillation parameters of *BMAL1,* **F** whereas the *KLF9* transcript levels remained relatively unchanged, and no period was detected. **G**, **H** MCF10A and **I**, **J** MDA-MB-231 cells stably overexpressing CLOCK were transiently transfected with a transient *BMAL1* expression vector prior to analysis of gene expression after 24 h. Forced expression of *CLOCK* and *BMAL1* induced the expression of **G**, **I** *KLF9* and **H**, **J** direct CLOCK/BMAL1 target *PER1* in both cell lines (Student’s *t-*test; *P* < 0.05). **K** MCF10A cells were transiently transfected with *CLOCK* and *BMAL1* expression vectors along with the eKSM luciferase reporter and *Renilla* luciferase normalization control. Ectopic expression of *CLOCK* and *BMAL1* induced eKSM luciferase activity relative to the pGL4.23 empty vector negative control lines (Student’s *t-*test; *P* < 0.001). Dashed lines indicate confidence bands demarcating the likely location of the true curve at 95% confidence level. Bars represent mean ± SEM with asterisks above the mean indicating significant differences. All experiments were performed with N ≥ 3 replicates

*KLF9* is expressed in a circadian fashion in the mouse liver [15], human skin [14], and mouse and human hippocampus [16]. To determine whether *KLF9* oscillates in the breast and recapitulate circadian gene expression in vitro, we synchronized mammary epithelial cell lines through incubation with 1 μM CORT pulse for 2 h after which cells were harvested every 4 h for 48 h [40]. In MCF10A cells, CORT pulse induced the rhythmic oscillation of *KLF9* mRNA (Fig. 3A-C, Additional file 7: Fig. S6A, B) and pre-mRNA (Additional file 7: Fig. S6C, D) that was antiphase with *BMAL1* transcript levels (Fig. 3A–C), with maximal *KLF9* mRNA and pre-mRNA levels occurring at around 24 h corresponding with the *BMAL1* mRNA nadir (Fig. 3A). Cosine wave regression analysis determined the period of *BMAL1* to be at 25.8 h (Fig. 3B), and 25.7–28.1 h for *KLF9* mRNA (Fig. 3C, Additional file 7: Fig. S6B) and pre-mRNA (Additional file 7: Fig. S6D). These generally agreed with the cycling parameters determined by harmonic regression using the CircWave software (Additional file 1: Table S3).

This is likewise consistent with in vivo time-series transcriptomic data from Yang and colleagues [54] (E-MTAB-5330) where the mouse *Klf9* gene was included in the list of genes with circadian expression. *Klf9* expression in the mouse mammary gland is indeed rhythmic, peaking at circadian time (CT) 11 and CT35 (Additional file 7: Fig. S6E, F,). As details on the period, amplitude, and phase shift were unavailable in the study by Yang et al. [54], we applied the same cosine-wave fit analysis on their microarray data and predicted the period to be 26.14 h (Additional file 7: Fig. S6F). The BMAL1 target genes *PER1*, *PER2*, and *DEC2* also exhibited an antiphase relationship of expression with that of the master clock regulator in normal MCF10A cells (Additional file 8: Fig. S7A–F; and Additional file 1: Table S3).

We employed the CORT pulse method to synchronize MDA-MB-231 cells (Fig. 3D–F). Oscillation of the *BMAL1* transcript in this cell line is still evident with the period determined to be 27.43 h (Fig. 3D, E), although expression did not decrease after the expected peak at t = 36 h as observed in MCF10A cells. While *BMAL1* mRNA had rhythmic expression in MDA-MD-231 similar to that in MCF10A, circadian oscillation of *KLF9* mRNA was absent in this BCa line (Fig. 3D, F). After decreasing at the 4-h timepoint after CORT withdrawal, *KLF9* transcript levels remained stable until t = 40 h and thereafter abruptly increased to baseline levels by the 48-h timepoint. Cosine wave fitting failed to detect any period for the *KLF9* time-series dataset in MDA-MB-231 cells (Fig. 3F) and CircWave analysis determined a much lower CircWave curve fit *p*-value and *r*^*2*^, as well as a much lower 40% amplitude compared to 100% amplitude determined for *KLF9* mRNA oscillation in MCF10A (Additional file 1: Table S3). *PER1* oscillation was likewise slightly irregular in the MDA-MB-231 line, with period determined to be 21.4 h (Additional file 8: Fig. S7G, H; and Additional file 1: Table S3).

Previous studies showed that *KLF9* can be induced by CLOCK/BMAL1 in keratinocytes and neuronal cells through multiple E-boxes upstream of its TSS [14, 16]. To determine if CLOCK/BMAL1 can influence *KLF9* gene transcription in mammary epithelial cell lines*,* we transiently overexpressed *BMAL1* in MCF10A and MDA-MB-231 cells stably transduced with *CLOCK* expression cassette and evaluated changes in *KLF9* expression. Forced expression of CLOCK and BMAL1 increased the levels of *KLF9* mRNA, and the established CLOCK/BMAL1 transcriptional target *PER1* mRNA in MCF10A (Fig. 3G, H) and MDA-MB-231 cells (Fig. 3I, J). To then evaluate whether the eKSM, which contains multiple E-boxes, can support CLOCK/BMAL1-dependent transactivation of the *KLF9* gene, we co-transfected the eKSM luciferase reporter plus *CLOCK* and *BMAL1* expression vectors into MCF10A cells. Forced expression of CLOCK and BMAL1 increased eKSM-driven luciferase activity (Fig. 3K).

### Feedback of KLF9 to the breast circadian oscillator

**Fig. 4:**
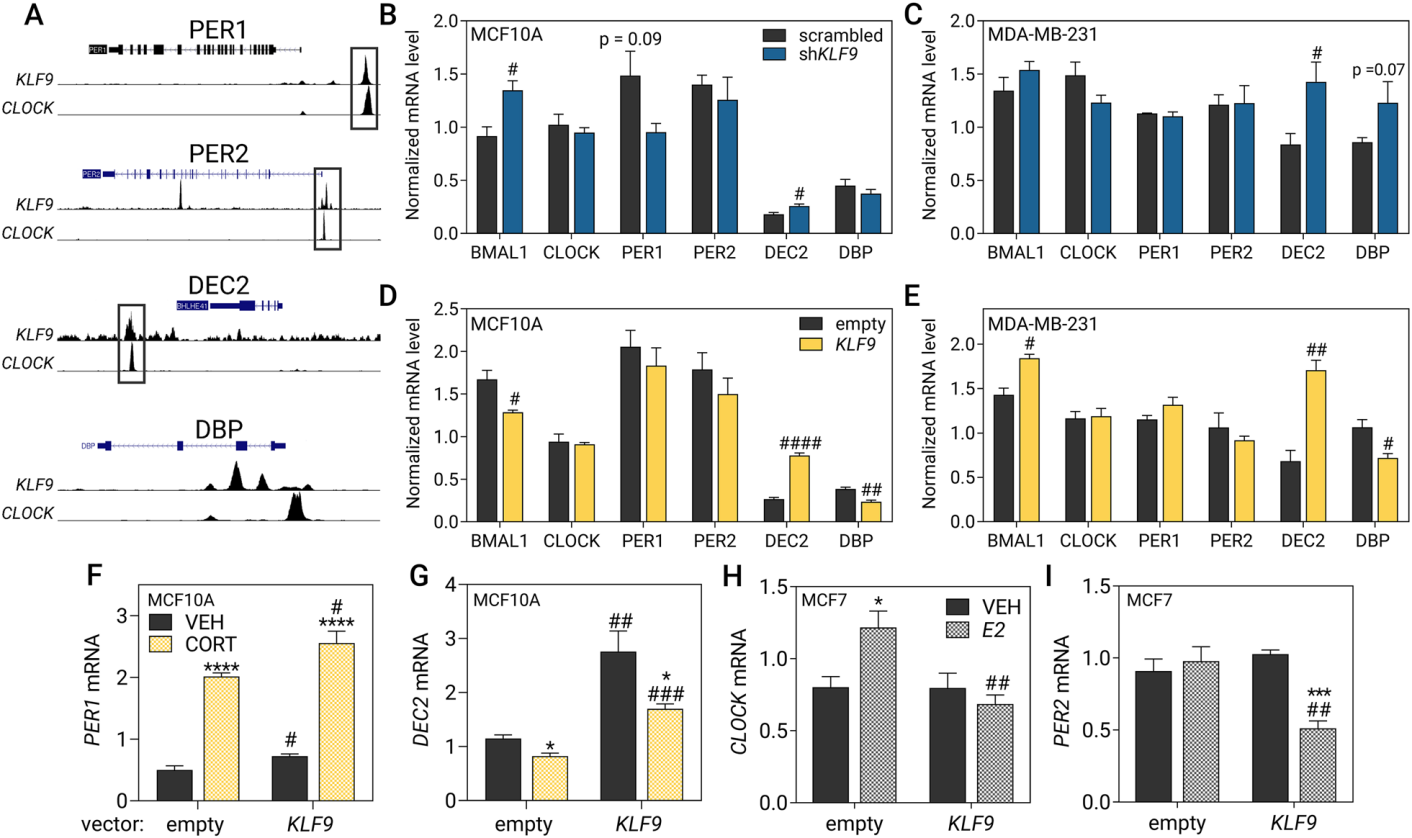
KLF9 influences the hormone response of some clock genes. **A** Publicly available KLF9 and CLOCK ChIP-seq data in MCF7 cells [47] were visualized using the UCSC genome browser [50] mapped to the human GRCh37/hg19 genome assembly. In black-outlined boxes are KLF9 peaks which often co-localize with CLOCK. Effects of *KLF9* **B**, **C** knockdown and **D**, **E** overexpression induced moderate changes on the expression the clock genes *BMAL1, CLOCK, PER1, PER2, DEC2,* and *DBP* in **B**, **D** MCF10A and **C**, **E** MDA-MB-231 cells as measured through RT-qPCR. **F**, **G** MCF10A cells overexpressing *KLF9* were treated with 100 nM CORT for 2 h prior to analysis of gene expression through RT-qPCR. *KLF9* overexpression augmented the **F** upregulation of *PER1* (two-way ANOVA; treatment: *P* < 0.0001; overexpression: *P* = 0.0027) and **G** repression of *DEC2* mRNA upon CORT treatment (two-way ANOVA; treatment: *P* = 0.0009; overexpression: *P* < 0.0001). **H**, **I** MCF7 cells overexpressing *KLF9* were treated with 1 μM E2 for 24 h prior to analysis of gene expression through RT-qPCR. Ectopic expression of *KLF9* abrogated the induction of **H** *CLOCK* (two-way ANOVA; treatment: *P* = 0.2205; Overexpression: *P* = 0.0153) and reduced *PER2* transcript in E2-treated cells (two-way ANOVA; Treatment: *P* = 0.0049; Overexpression: *P* = 0.0165). Bars represent mean ± SEM with statistically significant differences determined through two-way ANOVA for main effects of treatment or overexpression and Student’s *t-*test for individual effects of hormone treatment (^*^) and knockdown or overexpression (^#^). All experiments were performed with N ≥ 3 replicates

KLF9 has been previously demonstrated to co-localize with the core circadian regulator CLOCK on promoters of core clock and clock output genes in mouse hippocampal neurons [16]. To determine if the phenomenon is conserved in mammary epithelia, we evaluated publicly available KLF9 and CLOCK ChIP-seq datasets in MCF7 as there were no available datasets for MCF10A cells. As with hippocampal neurons, we observed a similar pattern in MCF7 cells where we found overlapping KLF9 and CLOCK ChIP-seq peaks at the loci of core clock genes *PER1-3* (Fig. 4A, Additional file 9: Fig. S8) and *CRY1-2* (TTFL #1) (Additional file 9: Fig. S8), accessory loop genes *NR1D1-2* (TTFL #2) (Additional file 9: Fig. S8), and auto-regulatory loop genes *DEC2* (TTFL #3) (Fig. 4A), as well as the clock output gene *TEF* (Additional file 9: Fig. S8). We also observed prominent KLF9 peaks in *DEC1*, *WEE1* (Additional file 9: Fig. S8)*,* and *DBP* (Fig. 4A) although they did not overlap with a CLOCK peak at these loci.

With this, we further probed the regulatory impact of KLF9 on baseline expression of clock genes and their response to both GC and E2 signaling. To this end, we stably expressed *KLF9-*specific shRNAs and effectively reduced *KLF9* expression by 60–80% in the three model cell lines using sh*KLF9* type #3 (sh3, Additional file 10: Fig. S9A, C, E). Since knockdown upon transduction of sh*KLF9* #3 was consistently higher compared to sh*KLF9* #4, we opted to use the cells transduced with this shRNA type for downstream functional analyses. To complement our knockdown experiments, we also overexpressed *KLF9* coding sequence in all three cell lines (Additional file 10: Fig. S9B, D, F). Finally, to further ascertain that *KLF9* knockdown or overexpression was successful, we also evaluated the expression of known KLF9-repressed target genes *DBP* [16]*, MAPK11* [55]*,* and *MEX3A* [55]*,* all three of which were generally increased upon KLF9 knockdown or downregulated upon KLF9 overexpression in MCF10A (Additional file 11: Fig. S10A, B), MCF7 (Additional file 11: Fig. S10C, D), MDA-MB-231 cells (Additional file 11: Fig. S10E, F).

We then evaluated changes in the expression of components of the core clock machinery (*BMAL1*, *CLOCK*, *PER1/2/3*, *CRY1/2*, *NR1D1/2*, and *DEC1/2*) and three clock output genes (*WEE1*, *TEF*, *DBP*) upon knockdown or overexpression of *KLF9* in normal MCF10A cells and in the highly aggressive MDA-MB-231 cancer line. Genetic perturbation generally led to moderate changes in clock gene expression, with *KLF9* knockdown in MCF10A resulting in increased levels of mRNAs for *BMAL1* and *DEC2* (Fig. 4B), as well as *CRY2, NR1D2,* and *DEC1* (Additional file 12: Fig. S11A)*.* In the MDA-MB-231 line, *KLF9* knockdown led to a consistent upregulation of *DEC2* (Fig. 4C) and *CRY2* (Additional file 12: Fig. S11B), but not in the other genes studied. On the other hand, *KLF9* overexpression in MCF10A reduced *BMAL1* and *DBP* transcript levels (Fig. 4D), whereas other clock genes like *DEC2* (Fig. 4D)*, CRY1, NR1D1,* and *NR1D2* increased (Additional file 12: Fig. S11C). In MDA-MB-231 cells, overexpression of *KLF9* resulted in an increase in *BMAL1, DEC2* (Fig. 4E)*, CRY2, NR1D1,* and *TEF* mRNAs (Additional file 12: Fig. S11D), but reduced the levels of *DBP* (Fig. 4E) and *DEC1* (Additional file 12: Fig. S11D). Notably, *BMAL1*, which increased upon *KLF9* knockdown in MCF10A, was reduced upon forced expression in the line. In addition, *DBP* which is downregulated by KLF9 in mouse hippocampal neurons [16] was unchanged upon *KLF9* knockdown in MCF10A, but exhibited a trend of upregulation in MDA-MB-231 cells. Overexpression of *KLF9* led to the reduction in *DBP* transcript levels in both cell lines (Fig. 4D, E).

In line with evidence that hormones can modulate the local mammary circadian clock by directly impacting expression of some core clock genes, we also investigated whether KLF9 can alter the GC or E2 response of established hormone-regulated clock genes. We treated the *KLF9*-overexpressing MCF10A cells with 100 nM CORT for 2 h and evaluated changes in the expression of clock genes, *PER1* and *DEC2*, known to be induced and repressed by CORT, respectively [56, 57]. CORT treatment induced *PER1* mRNA, and *KLF9* overexpression augmented the CORT-mediated increase in *PER1* transcript (Fig. 4F). On the other hand, CORT treatment decreased *DEC2* mRNA levels by 20%, and repression was further enhanced by ectopic expression of *KLF9* by 31% relative to vehicle-treated cells (Fig. 4G).

In addition, since E2 can directly impact the expression of *CLOCK* [11] and *PER2* [10] in MCF7, we also investigated whether KLF9 can influence the E2-mediated induction of these genes in this cell line. We treated empty vector- and *KLF9*-overexpressing MCF7 cells with 1 μM E2 for 24 h. Remarkably, *KLF9* overexpression abrogated the E2-dependent induction of *CLOCK* (Fig. 4H) and diminished the magnitude of E2-mediated increase in expression of the direct ERα target *GREB1* (Additional file 13: Fig. S12), consistent with its demonstrated antagonism of ERα-mediated signaling [19]. The transcript levels of *PER2* were refractory to E2 treatment in MCF7 but decreased upon concomitant *KLF9* overexpression (Fig. 4I).

### Functional role of KLF9 in breast cancer pathogenesis

**Fig. 5:**
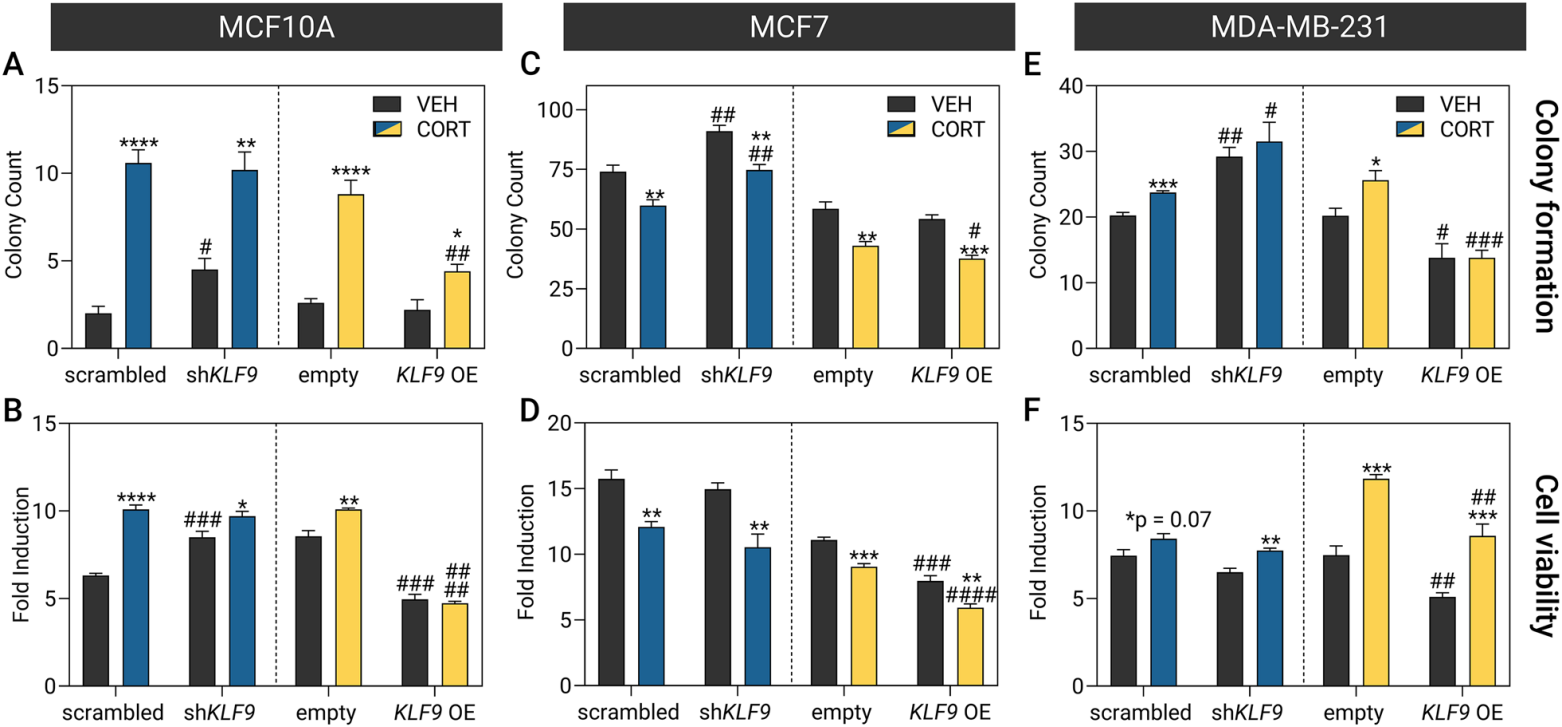
KLF9 restricts breast epithelial cell survival and proliferation. Effects of CORT treatment and *KLF9* knockdown (blue) or overexpression (yellow) on **A, C, E** cell survival and **B, D, F** viability of breast epithelial cell lines as evaluated using assays based on colony formation and resazurin reduction, respectively. The cells were treated with vehicle (100% ethanol) or CORT (100 nM) for 14 d and 96 h for the colony formation and cell viability assay, respectively. In MCF10A, CORT promoted **A** survival and **B** proliferation in both knockdown [2-way ANOVA; Colony formation: treatment: *P* < 0.0001; knockdown: *P* = 0.2060|Cell viability: treatment: *P* < 0.0001; Knockdown: *P* = 0.0004] and overexpression lines [2-way ANOVA; Colony formation: treatment: *P* < 0.0001; overexpression: *P* = 0.0005|Cell viability: treatment: *P* = 0.1045; overexpression: *P* < 0.0001. In the luminal MCF7 line, CORT treatment diminished **C** survival and **D** proliferation in both knockdown [2-way ANOVA; Colony formation: treatment, knockdown: *P* < 0.0001|Cell viability: treatment: *P* < 0.0001; knockdown: *P* = 0.0355] and overexpression lines [2-way ANOVA; colony formation: treatment: *P* < 0.0001; overexpression: *P* = 0.0269|Cell viability: treatment: *P* < 0.0001; overexpression: *P* < 0.0001]. Finally, in MDA-MB-231 cells, CORT treatment enhanced **E** colony formation and **F** proliferation in both *KLF9* knockdown [2-way ANOVA; colony formation: treatment, *P* = 0.1026; knockdown: *P* = 0.0002|Cell viability: treatment: *P* = 0.0006; Knockdown: *P* = 0.0057] and overexpression lines [2-way ANOVA; Colony formation: treatment: *P* = 0.0986; overexpression: *P* < 0.0001|Cell viability: treatment, overexpression: *P* < 0.0001]. Bars represent mean ± SEM with statistically significant differences determined through two-way ANOVA for main effects of treatment or overexpression and Student’s *t-*test for individual effects of hormone treatment (^*^) and knockdown or overexpression (^#^). All experiments were performed with N ≥ 4 replicates

To investigate the impact of the GC signaling and KLF9 in BCa etiology, we treated the *KLF9* knockdown and overexpression lines with CORT prior to functional analysis using assays of cancer hallmarks including survival, proliferation, apoptosis, and migration. The colony formation assay was utilized to determine the effects of CORT treatment and KLF9 on cellular survival and neoplastic transformation of breast epithelial cell models, while the resazurin-based assay that quantifies the changes in the bulk metabolism of the cells was used as an indirect measure of proliferation [58]. Non-malignant MCF10A cells formed very few colonies (Fig. 5A; Additional file 14: Fig. S13A, B). CORT treatment enhanced colony formation and proliferation (Fig. 5A, B; Additional file 14: Fig. S13G, H) in MCF10A, an effect that was unaffected by *KLF9* knockdown but diminished by *KLF9* overexpression. In the luminal MCF7 line, CORT instead reduced long-term cell survival (Fig. 5C; Additional file 14: Fig. S13C,D) and viability (Fig. 5D; Additional file 14: Fig. S13I, J). Knockdown of *KLF9* reduced the anti-survival effects of CORT but did not influence CORT-mediated inhibition of proliferation. In addition, *KLF9* overexpression consistently augmented the anti-tumorigenic effects of CORT in this cell line. For the aggressive TNBC MDA-MB-231line, CORT treatment generally enhanced survival (Fig. 5E; Additional file 14: Fig. S13E, F) and proliferation (Fig. 5F; Additional file 14: Fig. S13K, L) of the cells. Knockdown of *KLF9* enhanced survival but did not further affect proliferation. On the other hand, ectopic expression of *KLF9* reversed the pro-oncogenic effects of CORT.

**Fig. 6:**
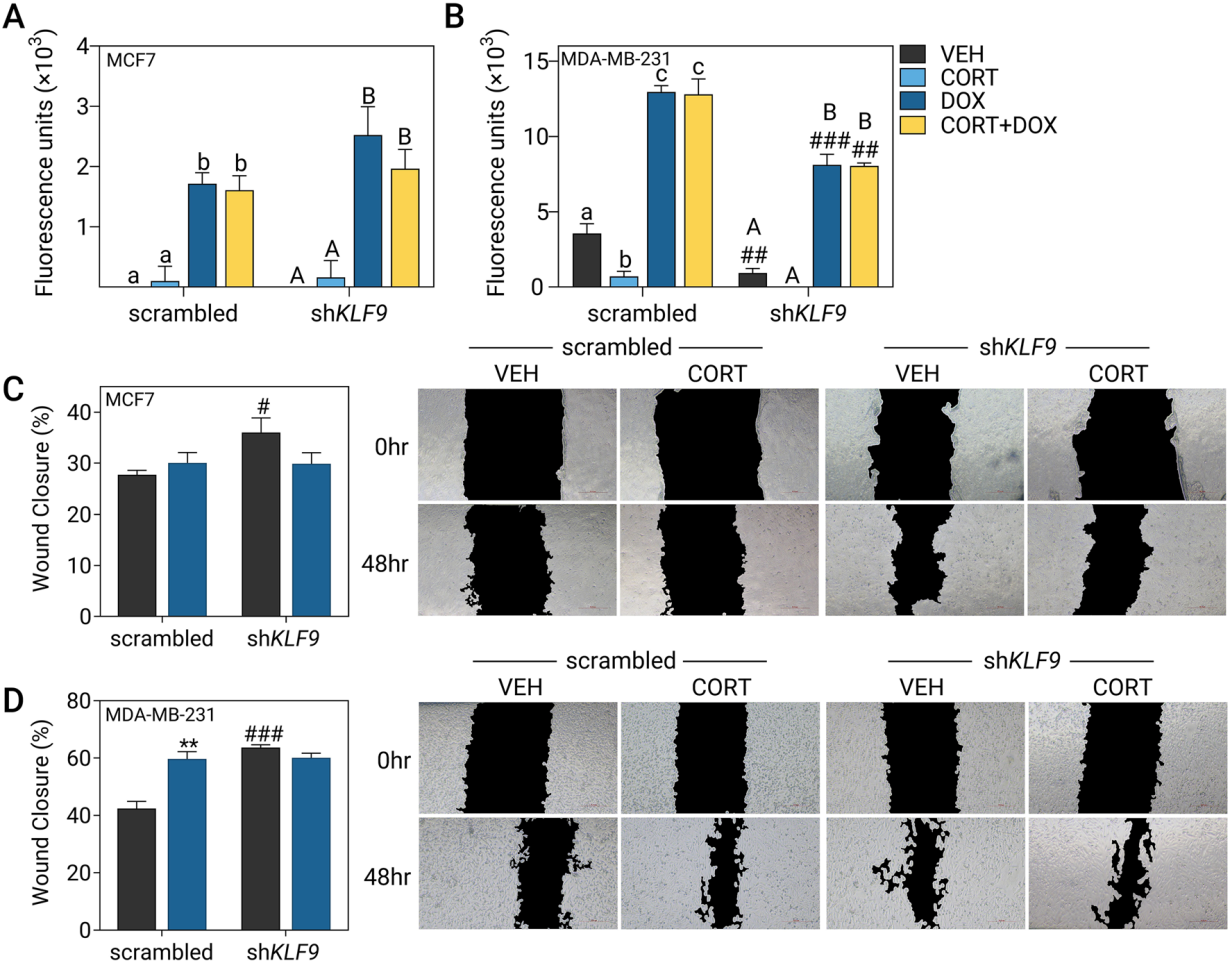
*KLF9* knockdown confers resistance to doxorubicin-induced apoptosis and induces migration in BCa cells. Apoptotic response of **A** MCF7 and **B** MDA-MB-231 cells was evaluated using an assay based on the activity of caspase-3 and caspase-7 to cleave a peptide conjugated to a DNA-binding dye. Scrambled shRNA- and sh*KLF9*-transduced cells were treated with CORT (100 nM), doxorubicin (DOX; MCF7: 1 μM, MDA-MB-231: 10 μM) or a combination of CORT and DOX for 24 h. CORT treatment did not influence DOX-induced apoptosis in (**A**) MCF7 (one-way ANOVA within shRNA type, *P* < 0.0001) and **B** MDA-MB-231 cells (one-way ANOVA; *P* < 0.0001). However, *KFL9* knockdown reduced the apoptotic signal in DOX-treated MDA-MB-231 cells (one-way ANOVA; *P* < 0.0001). Cell migration was assessed through a wound healing assay where cells were treated with vehicle (100% ethanol) or CORT (500 nM) and representative images of the scratch at each timepoint are shown to the right of each plot. **C** In MCF7, CORT treatment did not influence migration while *KLF9* knockdown slightly increased migration in vehicle-treated cells (two-way ANOVA; Treatment: *P* = 0.3935, Knockdown: *P* = 0.0767). **D** For MDA-MB-231, CORT treatment of scrambled shRNA-transduced cells promoted migration. *KLF9* knockdown likewise enhanced cell migration in vehicle-treated cells (2-way ANOVA; Treatment: *P* = 0.0015, Knockdown). Bars represent mean ± SEM with statistically significant differences determined through two-way ANOVA for main effects of treatment or overexpression and one-way ANOVA (effects of hormone treatment within an shRNA type followed by Tukey’s post-hoc test: *P* < 0.05; means with the same letter are not significantly different) or Student’s *t-*test for individual effects of hormone treatment (^*^) and knockdown (^#^). All experiments were performed with N ≥ 4 replicates

Next, we assessed the influence of KLF9 on DOX-induced apoptosis in the luminal MCF7 and aggressive TNBC MDA-MB-231 lines through a fluorescence-based assay that measures caspase-3/7 activation. DOX-mediated cytotoxicity in MCF7 cells was unaltered by CORT treatment and *KLF9* knockdown (Fig. 6A). In MDA-MB-231 cells, CORT treatment by itself protected against baseline apoptosis in both scrambled and sh*KLF9-*transduced cells. However, this protective effect was not apparent upon apoptotic induction by DOX. When *KLF9* was knocked down, however, the MDA-MB-231 cells were overall less susceptible to DOX-induced apoptosis (Fig. 6B).

Finally, the role of KLF9 in cell migration was evaluated through the scratch-wound assay. In MCF7 cells, CORT treatment did not affect migration while *KLF9* knockdown in vehicle-treated cells slightly enhanced wound closure rates (Fig. 6C). In MDA-MB-231 cells, CORT treatment augmented the migration rate of scrambled shRNA-transduced cells. *KLF9* knockdown resulted in a similar magnitude of increase in vehicle-treated cells but this was not further enhanced with CORT treatment (Fig. 6D).

## Discussion

The cellular circadian clock of the mammary gland is primarily driven by the intrinsic oscillatory expression of the components of the core clock machinery, and can be modulated by extrinsic factors like hormone signaling [9]. In particular, cellular timekeeping is directed by a core set of TFs, principally CLOCK and BMAL1, whose autoregulatory functions effect diurnal gene expression programs [3]. Dynamic regulation of clock-controlled genes is then tightly interlinked with hormone signaling, as GCs and E2 can alter expression of clock genes and serve as systemic cues to synchronize the local mammary gland clock to other regions in the body [11, 59–61]. As such, circadian desynchrony, brought about by either shiftwork or exogenous hormone intake, has been implicated in the neoplastic transformation in the breast [62, 63]. Maladaptive alterations to hormone signaling that is prevalent in BCa result in dysregulated expression of hormone-regulated clock genes, cascading onto the entire circadian molecular network owing to its interdependent nature [10, 11, 56, 59]. Disruption of the local mammary clock ultimately results in the aberrant circadian control of proliferation, metabolism, and invasive capacity, further exacerbating BCa progression [9, 64].

The *KLF9* gene encodes a TF rhythmically expressed in neurons, hepatocytes, and keratinocytes under the transcriptional control of CLOCK and BMAL1 [14–16]. It is likewise directly induced by GC signaling in different cellular contexts [14, 18, 52], and antagonizes E2-mediated transcriptional activity in endometrial epithelial cells [19]. Underscoring the emerging role KFL9 in hormone-responsive neoplasms, knockout of *Klf9* in mice increased overall endometrial carcinoma burden [65], while its ectopic expression promoted apoptosis in androgen-dependent prostate cancer cells [66] and restricted BCa metastatic spread [20]. Despite mounting evidence implicating KLF9 as a tumor suppressor, its role in the emerging link between hormone signaling, circadian disruption, and BCa development is yet to be explored. In this study, we investigated the mechanism behind the hormone and circadian regulation of the *KLF9* gene in the mammary gland and determined its reciprocal impact on the local mammary clock. We further evaluated the functional consequence of the genetic perturbation of *KLF9* on the oncogenic behavior of non-malignant breast, metastatic ER + , and aggressive TNBC cell models.

### *KLF9* is GC-inducible but E2-refractory in breast epithelia

**Fig. 7:**
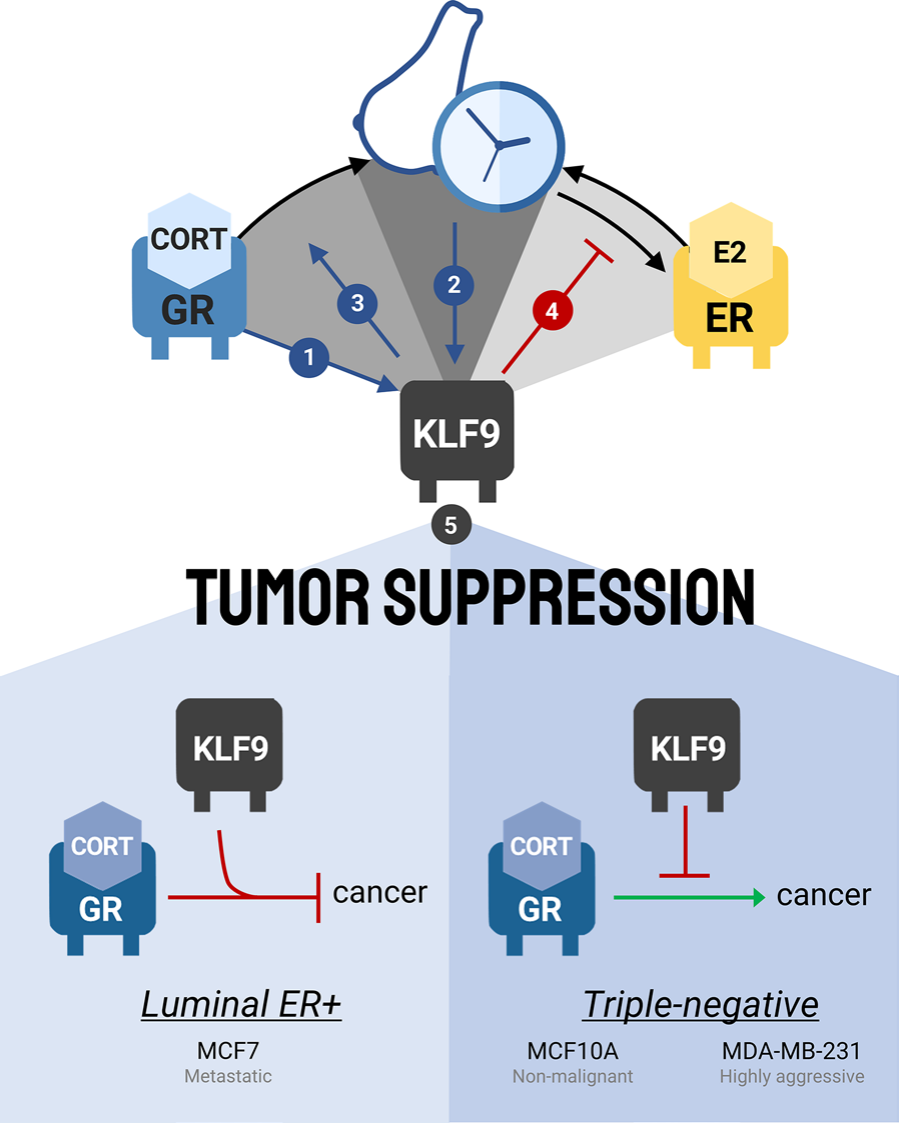
Circadian-hormone-*KLF9* crosstalk in BCa. The *KLF9* gene is coordinately regulated by GC (#1) and circadian (#2) signaling in normal breast epithelial cells. KLF9, in turn, feed backs to the breast circadian oscillator by modulating the GC (#3) and E2 (#4) response of core clock genes. In the context of BCa progression, KLF9 restricts oncogenesis in all mammary epithelial cell models used (#5). Moreover, in the luminal ER-expressing MCF7 cells, KLF9 enhanced the anti-tumorigenic effects of CORT signaling, while in triple-negative MCF10A and MDA-MB-231 cells, KLF9 restricted CORT-induced BCa progression

To investigate the hormone-dependent regulation of *KLF9* in the breast, we utilized three mammary epithelial cell models, namely MCF10A, MCF7, and MDA-MB-231, in order of increasing aggressiveness and metastatic potential. Notably, the pattern of *KLF9* downregulation in breast tumor samples was reflected by the cell models used, confirming the validity of these lines in recapitulating molecular events in vivo and further highlighting the potential anti-tumorigenic role of KLF9 in BCa. While *KLF9* is a direct transcriptional target of GR in triple-negative cell models regardless of metastatic status, luminal MCF7 cells do not appreciably increase *KLF9* expression in response to CORT, possibly owing to the lower expression of GR in the cell line. Taken together, these findings support direct GC action on the *KLF9* gene, corroborating evidence of *KLF9* being an immediate early gene upregulated in response to CORT in other tissues (Fig. 7, #1) [52, 67]. In contrast, *KLF9* gene expression was not responsive to E2 in the luminal MCF7 line.

Through in silico analysis of publicly available gene regulation datasets, we identified three candidate regulatory elements, namely the eKSM, aKSM, and KDE which contained at least one canonical GRE and exhibited CORT-mediated transcriptional activation in enhancer-reporter assays and in analyses of eRNA expression. Moreover, CORT-inducible eRNA production from all three regulatory regions was directly and specifically mediated by the GR in a similar manner to the *KLF9* mRNA. Thus, the aKSM, eKSM, and KDE may function in a context-dependent manner to coordinate GR-dependent induction of *KLF9,* and generation of local eRNAs that may strengthen the association of each enhancer with the TSS culminating in cell-type dependent or amplified *KLF9* expression levels—a similar regulatory logic recently elucidated in lung epithelial cells [18].

### *KLF9* is a circadian output gene in mammary epithelia

Expression of *KLF9* is circadian in the mouse mammary gland and in the synchronized human normal breast MCF10A cells (Fig. 7, #2). In addition, *KLF9* mRNA was antiphase with *BMAL1* expression—a pattern expected between the master clock regulator and a clock target gene [68] as seen in *PER1, PER2, and DEC2 *in our time-series dataset. Moreover, a previous study also revealed that the cycling phase of *PER1-3* and *NR1D1* in synchronized MCF10A cells are likewise delayed by approximately 12 h relative to *BMAL1* [69]. Our findings agree with the rhythmic oscillations of *Klf9* mRNA previously demonstrated in the hippocampus, liver, and keratinocytes [14–16]. In addition, around 30% of the members of the *Klf* family of TFs (*Klf9*, − 10, − 11, − 13, 15, − 16) show oscillatory transcript expression and are genomic targets of CLOCK + BMAL1 in the mouse liver [15]. Oscillation of *KLF9* may be mediated in part through cell-intrinsic CLOCK/BMAL1 transactivation, as evidenced by the upregulation in *KLF9* mRNA upon forced expression of CLOCK and BMAL1 and with CLOCK/BMAL1-mediated induction of *KLF9* from the canonical E-box located within the eKSM which is also observed in other cell lines [14, 16]. In addition to the local clock, extrinsic factors like systemic GC rhythms and temperature cycles have also been shown to contribute to *KLF9* oscillation in vivo [14].

In contrast, *KLF9* does not oscillate in entrained MDA-MB-231 cells and circadian expression of *BMAL1* and *PER1* was slightly irregular in the cancer cell line as well. Loss of rhythmic expression of clock genes is common in cancer, especially since the integrity of the circadian clock is important for tumor suppression in vivo. Aberrant cycling and expression of clock genes, including *PER1-3, CRY1-2,* and *NR1D1*, is prevalent in BCa cell lines and primary tumors [69–71] and may be caused by increased stromal stiffness in BCa tumors, given that the tension-sensing Rho/ROCK pathway modulates the activity of the core circadian complex [54, 70]. In turn, abnormal timing of core clock gene expression culminates in dysfunctional cellular readout since several genes that participate in mitotic licensing, nucleotide excision repair, and ECM remodeling are all under circadian control [8, 64, 72, 73]. Thus, the loss of circadian oscillation of *KLF9* in MDA-MB-231 cells may not only be a consequence of dysregulated rhythms in BCa but may also actively influence tumor progression depending on the transcriptional program orchestrated by KLF9 downstream of its rhythmic expression.

### KLF9 may act as a hormone-regulated feedback regulator of the local breast clock

As a TF, KLF9 may activate or repress transcription, depending on the chromatin context and interacting proteins [74]. While KLF9 is an established clock output gene [15, 75–77], the possibility that it may co-regulate CLOCK/BMAL1-controlled genes and modulate the expression of components of core clock machinery in breast epithelial cells remains under-explored. Interestingly, we found that KLF9 co-localizes with CLOCK within the genomic locale of several core clock and clock target genes in breast epithelial cells, parallel to what was observed in mouse hippocampal neurons [16]. With this, we screened for alterations to expression levels of core clock genes *BMAL1, CLOCK, PER1-3, CRY1-2, DEC1-2*, and clock output genes *DBP, TEF,* and *WEE1* upon *KLF9* knockdown or overexpression in MCF10A and MDA-MB-231 under baseline conditions (complete media). While genetic perturbation of *KLF9* led to moderate changes in gene expression of several clock genes, *DBP* decreased upon *KLF9* overexpression. In fact, in the mouse hippocampus, cyclic KLF9 association with an intronic enhancer within the *Dbp* locus repressed *Dbp* expression and partially directs the rhythmicity of the key clock output gene in vivo [16].

Given that KLF9 only moderately affects the baseline mRNA levels of some clock genes in mammary epithelial cell lines, we then investigated whether it could alter the hormone response of clock genes that are regulated by GC or E2. Forced expression of *KLF9* in serum-free media enhanced the basal transcription of the CORT-regulated clock genes *PER1* and *DEC2*. KLF9 enhanced CORT-induced *PER1* expression and CORT-mediated repression of *DEC2* (Fig. 7, #3). This is reminiscent of Klf9 being a key feedforward regulator of GR transcriptional activity in zebrafish, wherein it mediates upregulation of proinflammatory genes upon chronic cortisol exposure [78].

Consistent with its function as a negative regulator of ERα-mediated signaling, *KLF9* overexpression abrogated E2-induced upregulation of the direct ERα target *GREB1* and the core circadian TF *CLOCK* (Fig. 7, #4). This may be mediated through direct sequestration of the ERα co-activator Sp1 which precludes it from binding to the promoter of E2-induced genes [19] or through competitive binding of KLF9 to non-canonical Sp1 motifs [55]. Remarkably, we also observed a decrease in the levels of *PER2* mRNA upon concomitant E2 treatment and *KLF9* overexpression. As such, KLF9 antagonism of E2 signaling may be one of the mechanisms by which GR signaling negatively regulates E2 transcriptional activity [79] and more importantly, this represents another mechanism by which KLF9 may alter cycling dynamics of the local circadian oscillator in the breast and possibly affect BCa initiation and progression.

### KLF9 restricts breast cancer tumorigenesis

Reciprocal contributions of dysregulated E2 and GC signaling towards circadian disruption have long been established [80], and the resulting dysregulation of the tightly interdependent regulatory network in BCa contribute to overall pathogenesis [9]. Estrogens and ERα are considered to be the primary drivers of BCa initiation and progression [79], while GC signaling plays a positive or negative role in BCa largely depending on the molecular landscape. In particular, high GR expression is associated with increased survival in ER + BCa patients; whereas, the converse is true for patients with TNBC [12]. Metastatic BCa patients have elevated plasma cortisol concentrations relative to their early-stage BCa counterparts [81], and normal ultradian fluctuations in cortisol concentrations suggest good prognosis in metastatic BCa patients [13]. As such, elevated serum cortisol consequent to disrupted biological clocks in patients with ER– BCa may confer pro-tumorigenic effects in terms of neoplastic transformation, colonization of distant metastatic sites, and resistance to chemotherapy [82, 83]. Remarkably, a recent study has revealed that proliferation and intravasation of BCa circulating tumor cells is driven by the circadian rhythm through a GC-regulated pathway [84].

With the link between dysregulated hormone signaling, circadian disruption, and BCa progression, we then determined the contextual role of KLF9 in the hormone-circadian regulatory axis towards BCa progression through several cancer hallmark assays. We treated the three breast epithelial cell models with CORT to assess whether KLF9 influenced the differential effects induced by CORT in ER + BCa cells relative to their ER– counterparts. In ER + BCa cells, liganded GR is known to suppress ER chromatin occupancy at ER-regulated enhancers of cell-cycle genes, thereby inhibiting E2-induced proliferation in luminal BCa [85]. In triple-negative breast epithelia, CORT induces the expression of genes involved in proliferation, cell survival [86], and metastasis [82, 87]. Consistent with associated studies [12, 88–90], we found that CORT treatment conferred pro-tumorigenic effects in ER– MCF10A and MDA-MB-231 cells while the reverse is observed for luminal ER + MCF7 cells. Moreover, our cellular assays demonstrate that KLF9 generally functions as a tumor suppressor in mammary epithelial cells by inhibiting neoplastic transformation, reducing proliferation, enhancing DOX-induced apoptosis, and restricting migration (Fig. 7, #5). In MCF10A and MDA-MB-231, KLF9 rescued the pro-survival and pro-proliferative effects conferred by CORT treatment while it potentiated the anti-tumorigenic effects of CORT in luminal MCF7 cells. KLF9 may also cooperate with GR to antagonize E2-induced proliferation in a mechanism reminiscent of its antagonistic activity on ERα-mediated transcription [19]. Moreover, KLF9 also sensitized MDA-MB-231 cells to DOX-induced apoptosis, likely mediated through a similar KLF9-dependent upregulation of pro-apoptotic NOXA in multiple myeloma cells [91] and yet other unidentified factors mediating response to genotoxic stress.

## Conclusions

In summary, we demonstrate the mechanism behind hormone and circadian regulation of *KLF9* and the functional impact of this axis towards BCa progression. To our knowledge, this study is the first to provide evidence for KLF9 in the consequential link between the hormone and circadian signaling networks towards BCa pathogenesis. Aberrant *KLF9* expression and oscillation in BCa may disrupt the normal cycling dynamics of the entire circadian network as KLF9 can influence the hormone response of core cellular clock and clock output genes, ultimately resulting in tumorigenesis. Our study marks a necessary first step in filling the crucial gap in knowledge of the key players in hormone-associated circadian disruption and BCa etiology.

## Supplementary Information

**Additional file 1: **Krüppel-like factor 9 (KLF9) links hormone dysregulation and circadian disruption to breast cancer pathogenesis. Additional file 1 contains additional file material including additional tables (**Tables S1**–**3**) and additional figure legends (**Figure S1**–**13**).

**Additional file 2: Figure S1.** Baseline *NR3C1* (GR) expression across three breast epithelial lines. MCF7 expressed considerably lower levels of GR mRNA relative to its triple-negative counterparts, MCF10A and MDA-MB-231 (one-way ANOVA; *P* < 0.0001).

**Additional file 3: Figure S2.** Expression of *KLF9* and the direct ER target *GREB1* upon estrogen treatment. Changes in *KLF9 *transcript levels in response to 10 nM E2 treatment from (39) were plotted as TPM over time. **(A)** Induction of *KLF9 *by E2 at 30 min abruptly decreased to baseline by 1 hr and continued to decline slowly over time. **(B)** *GREB1 *which is directly upregulated by ER signaling served as positive control. **(C) **In MCF7 cells treated with increasing doses of E2, *GREB1* mRNA was induced starting at 10 nM E2 (one-way ANOVA; *P *< 0.0001).

**Additional file 4: Figure S3.** GR localization in the *KLF9* proximal promoter, eKSM and aKSM. The UCSC genome browser (47) was used to visualize the *KLF9 *locus and surrounding non-coding regions mapped to the human GRCh37/hg19 genome assembly. Highlighted are the proximal promoter (3kb upstream of TSS; green), eKSM (blue), and aKSM (yellow).

**Additional file 5: Figure S4.** Transcription factor response elements in the eKSM, aKSM, and KDE. In each of the enhancers, LASAGNA search 2.0 (48) was used to identify GREs and EREs, while CLOCK-binding sites were manually determined based on previously derived sequences in order of decreasing induction by CLOCK in a reporter enhancer assay as previously described (EboxA = CACGTG, EboxB = CACGTT or AACGTG EboxC = CACGCG, EboxE = CACGAG) (15).

**Additional file 6: Figure S5.** CORT-dependent transcription of enhancer RNAs (eRNA) from the aKSM, eKSM, and KDE is a direct effect of GR activity. MCF10A cells were pre-incubated with 100 μg/mL CHX for 30 min or 1 μM MIF before treatment with 300 nM CORT for 2 hr. **(A-F)** Nascent enhancer RNA transcription at basal conditions can be detected from all three *KLF9* enhancer regions: eKSM, aKSM, and KDE. **(A, C, E)** CORT treatment significantly induced eRNA transcription at all three regions that is not altered in the presence of the protein synthesis inhibitor CHX (Student’s *t*-test; *P* < 0.001). **(B, D, E)** CORT-dependent transcription of the eRNAs is GR-specific as pre-incubation with the GR-selective antagonist MIF abolished the increase in eRNA transcript (one-way ANOVA; eKSM: *P *< 0.0001; aKSM: P = 0.0007; KDE: P = 0.0012).

**Additional file 7: Figure S6.** *KLF9* transcript time-course expression in human cell lines and *in vivo* murine mammary epithelia. **(A-D)** MCF10A cells were pulsed with 1 μM CORT for 2 hr to synchronize circadian gene expression prior to collection of RNA every 4 hr. Circadian expression of both *KLF9* **(A, B) **mRNA and **(C, D) **pre-mRNA (blue lines) was antiphase with the expression of *BMAL1* (black) both peaking at 24 hr concurrent with *BMAL1* expression nadir. **(A, B) **Expression of *KLF9 *mRNA, assayed through another primer set in RT-qPCR, was still rhythmic with period determined to be 25.68 hr. **(C, D)** This is consistent with the oscillation of *KLF9 *pre-mRNA with period calculated to be 26.47 hr. (**E, F**) Transcriptome analysis of the mouse breast circadian clock was performed by obtaining time-series microarray data from Yang et al. (54) where mammary tissues were isolated every 4 hr for 48 hr from mice kept under total darkness.

**Additional file 8: Figure S7.** Circadian expression of *PER1*, *PER2, *and *DEC2 *mRNA in MCF10A cells and *PER1 in* MDA-MB-231 cells. **(A-F)** MCF10A and **(G-H) **MDA-MB-231 cells were pulsed with 1 μM CORT for 2 hr to synchronize circadian gene expression prior to collection of RNA every 4 hr. Circadian expression of BMAL1 target genes (blue) **(A, B) ***PER1, ***(C, D) ***PER2, *and **(E, F) ***DEC2 *was antiphase with the expression of *BMAL1 *(black), with maximal target transcript levels occurring at *BMAL1* nadir (t = 24 hr). (**B, D, F**) All *BMAL1* targets have periods around 24 hr as determined through cosine-wave regression analyses. **(G) **The antiphase oscillation of *BMAL1* and *PER1 *is conserved in the MDA-MB-231 line. However, rhythmic oscillation of both genes is slightly aberrant, with *BMAL1* expression plateauing at t = 36 hr and **(H) ***PER1 *abruptly increasing at t = 48 hr in contrast to what is observed in MCF10A.

**Additional file 9: Figure S8.** Overlap in KLF9 and CLOCK binding to genomic loci of core clock and clock target genes. Publicly available KLF9 (GSE105301) and CLOCK (GSE127640) ChIP-seq data in MCF7 cells (47) were obtained from Gene Expression Omnibus and visualized using the UCSC genome browser (50) . Clock gene loci and surrounding non-coding regions were mapped to the human February 2009 (GRCh37/hg19) genome assembly. In black-outlined boxes are KLF9 peaks which almost always co-localize with CLOCK in the same cell line.

**Additional file 10: Figure S9.** Validation of *KLF9* knockdown and overexpression in mammary epithelial cell models. **(A, C, E)** Human *KLF9 *was knocked down in MCF10A, MCF7, and MDA-MB-231 via lentiviral-mediated transduction of one of two *KLF9 *shRNAs (shKLF9-3: TRCN0000013630; shKLF9-4: TRCN0000013631). *KLF9 *expression was consistently reduced between scrambled and knockdown cells in each cell line for shKLF9-3 (Student’s *t-*test; MCF10A: *P* < 0.0001; MCF7: *P *= 0.0001; MDA-MB-231: *P* < 0.0001), but not shKLF9-4 (Student’s *t-*test; MCF10A: *P* = 0.0419; MCF7: *P = *0.7385; MDA-MB-231: *P *= 0.0077). **(B, D, F)** *KLF9* was successfully overexpressed in all three epithelial cell models (Student’s *t-*test; MCF10A: *P *< 0.0001; MCF7: *P *< 0.0001; MDA-MB-231: *P* < 0.0001).

**Additional file 11: Figure S10.** Validation of KLF9 targets upon *KLF9 *knockdown or overexpression in three breast epithelial cell lines. Effects of *KLF9* **(A, C, E)** knockdown and **(B, D, E) **overexpression on the expression of established KLF9*-*repressed targets *DBP *(16)*, MAPK11 *(55)*, *and *MEX3A *(55) in **(A, C) **MCF10A, **(B, D) **MCF7, and **(E, F) **MDA-MB-231 cells as measured through RT-qPCR. In MCF10A, **(A) **knockdown of *KLF9 *increased the expression of *MAPK11 *and *MEX3A *mRNA (Student’s *t-*test; *MAPK11: P = *0.0155, *MEX3A: P = *0.0082), **(B)** whereas overexpression conferred the converse effect of downregulating the expression of *DBP, MAPK11, *and *MEX3A *transcript levels (Student’s *t-*test; *DBP: P = *0.0047, *MAPK11: P = *0.0261, *MEX3A: P = *0.0054). **(C) **Knockdown of *KLF9 *in MCF7 cells led to a significant increase in *DBP *expression, as well a trend in upregulation of *MAPK11 *mRNA (Student’s *t-*test; *DBP: P = *0.0009, *MAPK11: P = *0.1104). **(D) **This is consistent with the observed decrease in *MAPK11 *and trend of downregulation in *DBP *transcript levels upon *KLF9 *overexpression (Student’s *t-*test; *DBP: P = *0.0927, *MAPK11: P = *0.0349). **(E) **These were generally consistent in MDA-MB-231 cells, with *KLF9 *knockdown resulting in increased *MAPK11 *mRNA levels and a trend of upregulation in *DBP *transcript (Student’s *t-*test; *DBP: P = *0.0788, *MAPK11: P = *0.0001), while overexpression concordantly downregulated all three *KLF9*-downregulated targets (Student’s *t-*test; *DBP: P = *0.0196,* MAPK11: P = *0.0001, *MEX3A: P = *0.0041).

**Additional file 12: Figure S11.** Genetic perturbation of *KLF9 *moderately influences expression of other clock genes. Effects of *KLF9 ***(A, B)** knockdown and **(C, D)** overexpression on the expression of core clock and clock output genes in **(A, C)** MCF10A and **(B, D)** MDA-MB-231 cells as measured through RT-qPCR. *KLF9* knockdown resulted in a significant increase in expression of *CRY2, NR1D2,* and* DEC1* in MCF10A cells (Student’s *t* -test; *CRY2*: *P = *0.0225, *NR1D2*: *P = *0.0322, *DEC1*: *P* = 0.0012). **(B)** The increase is consistent for *CRY2* in MDA-MB-231 *KLF9 *knockdown cells (Student’s *t*-test; *CRY2*: *P* = 0.0136). **(C)** On the other hand, overexpression of *KLF9* in MCF10A led to an increase in *CRY1, NR1D1, NR1D2* (Student’s *t-*test; *CRY1*: *P = *0.0374, *NR1D1*: *P = *0.0013, *NR1D2*: *P = *0.0363). **(D)** This was mostly consistent in MDA-MB-231 cells except for an observed increase in *CRY2 *and *TEF* and a decrease in *DEC1* transcript levels (Student’s *t*-test; *CRY2*: *P = *0.0005, *NR1D1*: *P *= 0.0056, *DEC1*: *P = *0.0091, *TEF*: *P = *0.0215).

**Additional file 13: Figure S12.** Overexpression of *KLF9 *abrogates E2 induction of *GREB1 *in ER+ MCF7 cells. MCF7 cells were treated with 1 μM E2 for 24 hr prior to analysis of gene expression. *GREB1* mRNA was induced upon E2 treatment in empty vector control MCF7 cells while *KLF9 *overexpression attenuated the induction (two-way ANOVA; Treatment: *P *< 0.0001; Overexpression: *P *= 0.0020; Interaction: *P *= 0.0057).

**Additional file 14: Figure S13.** Effects of *KLF9* knockdown or overexpression and CORT treatment on colony formation and viability of breast epithelial cells. Cell survival **(A-F)** and viability **(G-L)** were assessed in *KLF9* **(A, C, E, G, I, K)** -knockdown and **(B, D, F, H, J, L)** -overexpressing cells treated with either vehicle or CORT (100 nM) using the colony formation and resazurin reduction assays, respectively. For the colony formation assay, representative images of colonies of **(A, B)** MCF10A, **(C, D)** MCF7, and **(E, F)** MDA-MB-231 cells stained with crystal violet after 14-day treatment. For the cell viability assay, CORT treatment promoted cell proliferation in **(G, H)** MCF10A and **(K, L)** MDA-MB-231 cells, but had opposite effects in **(I, J) **MCF7 cells.

## Data Availability

All data generated or analyzed during this study are included in this published article and its supplementary information files.

## Abbreviations

BCa: Breast cancer
E2: 17β-Estradiol
GC: Glucocorticoid
KLF9: Krüppel-like factor 9
TTFL: Transcriptional-translational feedback loop
CLOCK: Circadian locomotor output cycles kaput
BMAL1: Brain and muscle ARNTL-like 1
PER: Period
CRY: Cryptochrome
NR1D1/2: Nuclear receptor subfamily 1 group D1/2
ER: Estrogen receptor
GR: Glucocorticoid receptor
TF: Transcription factor
PR: Progesterone receptor
HER2: Human epidermal growth factor receptor 2
TNBC: Triple negative BCa
CS: Charcoal-stripped
HS: Horse serum
FBS: Fetal bovine serum
KSM: Klf9 synergy module
eKSM: Extended KSM
aKSM: Adjacent KSM
KDE: KLF9 distal enhancer
TSS: Transcription start site
PCR: Polymerase chain reaction
ORF: Open reading frame
GTEx: Genotype-Tissue Expression Project
TCGA: The Cancer Genome Atlas
GEPIA: Gene Expression Profiling Interactive Analysis
SCAN-B: Sweden Cancerome Analysis Network-Breast
TPM: Transcript per million reads
CORT: Hydrocortisone or cortisol
CHX: Cycloheximide
MIF: Mifepristone
eRNA: Enhancer RNA
GRE: GC response element
ERE: E2 response element
DOX: Doxorubicin
EDE: ERRFI1 downstream enhancer
DEX: Dexamethasone
