# Supplementary material for: Krüppel-like factor 9 (KLF9) links hormone dysregulation and circadian disruption to breast cancer pathogenesis
Source: Cancer Cell Int. 2023 Feb 23;23:33. doi: 10.1186/s12935-023-02874-1 (PMC9948451; doi:10.1186/s12935-023-02874-1)
Supplement: Supplementary file 1 — Additional file 1: Krüppel-like factor 9 (KLF9) links hormone dysregulation and circadian disruption to breast cancer pathogenesis. Additional file 1 contains additional file material including additional tables (Tables S1–3) and additional figure legends (Figure S1–13). [file 12935_2023_2874_MOESM1_ESM.docx]

**ADDITIONAL FILE 1**

**Krüppel-like factor 9 (KLF9) links hormone dysregulation and circadian disruption**

**to breast cancer pathogenesis**

Weand S. Ybañez and Pia D. Bagamasbad^#^

National Institute of Molecular Biology and Biotechnology, University of the Philippines Diliman, Quezon City, Philippines

Short title: KLF9 in the hormone-circadian axis in breast cancer

^#^Address all correspondence to:

Pia D. Bagamasbad, PhD

National Institute of Molecular Biology and Biotechnology

National Science Complex, University of the Philippines

Diliman, Quezon City, Metro Manila 1101

Philippines

Tel: +632-8981-8619

Fax: +632-8925-3250

Email: pdbagamasbad@up.edu.ph

**Additional Tables**

**Table S1. Oligonucleotide primers used to amplify or anneal genomic, complementary, and short hairpin DNA for subcloning**

| **Target** | **Forward** | **Reverse** |
| --- | --- | --- |
| aKSM | 5’-ATTGGTACCCGAGGTTATG  CGAGGTAAC-3’ | 5’-ATCAAGCTTTAGGAAACAT  TCCTGCCATTCC-3’ |
| eKSM | 5’-ATAGGTACCATCTCGACAC  CCTACTCTGC-3’ | 5’-CGTAAGCTTGGAGGATAA  GTTTAGCTCGCAG-3’ |
| KDE | 5’-ATTGGTACCTTCCAAAGTT  GATGCCAAGTC-3’ | 5’-GCGAAGCTTAAACAACTTT  CTTGACCCTCTG-3’ |
| CLOCK Ext | 5’- TCTGAGACTTATGGTTG  G-3’ | 5’-GTCATTTCATAGCTGAG  C-3’ |
| CLOCK Int | 5’-TTTGCGATCGCTATGTTGTT  TACCGTAAGCTGTAG-3’ | 5’-TGAACGCGTCTGTGGTTGA  ACCTTGGAAG-3’ |
| BMAL1 Ext | 5’-GTGTAAGAACTGTGACTTC  AG-3’ | 5’-TTCTCTCCTTATCCAGTAA  GC-3’ |
| BMAL1 Int | 5’ -TTTGCGATCGCAATGGCAG  ACCAGAGAATGG-3’ | 5’-GCGACGCGTTAGTGTTATC  AGCGGCCATG-3’ |
| KLF9 Ext | 5’-CAGTCGAATAAACTTGCGA  C-3’ | 5’-AGTGTTGTTGACTTTGATC  TTAG-3’ |
| KLF9 Int | 5’-TTTGCGATCGCATGTCCGC  GGCCGCCTAC-3’ | 5’-TGCGACGCGTACCTAACA  AAGCGTTGGCCAGC-3’ |
| shKLF9-3  (TRCN0000013630) | 5’-CCGGCTCCCATCTCAAAGC  CCATTACTCGAGTAATGGGCTTTGAGATGGGAGTTTTTG-3’ | 5’-AATTCAAAAACTCCCATCT  CAAAGCCCATTACTCGAGTAATGGGCTTTGAGATGGGAG-3’ |
| shKLF9-4  (TRCN0000013631) | 5’- CCGGAGCATGATCAAGCG  ATCGAAACTCGAGTTTCGATCGCTTGATCATGCTTTTTTG-3’ | 5’- AATTCAAAAAAGCATGAT  CAAGCGATCGAAACTCGAGTTTCGATCGCTTGATCATGCT-3’ |

**Table S2. Oligonucleotide primers for RT-qPCR**

| **Target** | **Forward** | **Reverse** |
| --- | --- | --- |
| *18S rRNA* | 5’-GGATGTAAAGGATGGAAA  ATACA-3’ | 5’-TCCAGGTCTTCACGGAGCT  TGTT-3’ |
| *GAPDH* | 5’-TGCACCACCAACTGCTTAG  C-3’ | 5’-GGCATGGACTGTGGTCAT  GAG-3’ |
| *KLF9* mRNA (pair #1) | 5’-TGGCTGTGGGAAAGTCTAT  G-3’ | 5’-GTCTGAGCGGGAGAACTTT  T-3’ |
| *KLF9* mRNA (pair #2) | 5’-CAGAGTGCATACAGGTGAA  CGG-3’ | 5’-TTCTCACACAGCGGACAG  C-3’ |
| *KLF9* pre-mRNA | 5’-TCCCATCTCAAAGCCCATT  AC-3’ | 5’-CATGTTTGCACCCTTTCG  G-3’ |
| *CLOCK* mRNA | 5’-CACAAGGCATGTCCCAGTT  TC-3' | 5’-ATCATGCGTGTCCGTTGTT  C-3' |
| *BMAL1* mRNA  (*ARNTL*) | 5’-CGCTTTGAGGTGACCAAGT  C-3' | 5’-TGCCATTGGATGATCTGAA  GTC-3' |
| *PER1* mRNA | 5’-TGCCTCCTCCTCCTCCTATA  C-3' | 5’-CTGACGGCGGATCTTTCTT  G-3' |
| *PER2* mRNA | 5’-CATCGACGTGGCAGAATGT  G-3' | 5’-GCTGAGTCCCAGAGAAGG  AATATC-3' |
| *PER3* mRNA | 5’-CCGAATGGTGGTGGTGAAT  G-3' | 5’-GTGCTCATCGTTCCTCAAA  TCC-3' |
| *CRY1* mRNA | 5’-CACCTGTTGAAGCAAGGA  AGAAG-3' | 5’-TGCTCTGTCTCTGGACTTT  AGG-3' |
| *CRY2* mRNA | 5’-GGGACTACATCAGGCGATA  CC-3' | 5’-TGCTGGTAAATCTGCTTCA  TTCG-3' |
| *NR1D1* mRNA  (REV-ERBα) | 5’-GAGTCAAGGTCCAGTTTGA  ATGAC-3' | 5’-TGATGACGCCACCTGTGTT  G-3' |
| *NR1D2* mRNA  (REV-ERBβ) | 5’-GCGAAGGCTGTAAGGGTTT  C-3' | 5’-TGAAGCGACATTGCTGACA  TC-3' |
| *BHLHE40* mRNA (DEC1) | 5’-ATAAAGCGGAGCGAGGAC  AG-3' | 5’-TCCAAGTGACCCAAAGTTG  TAAG-3' |
| *BHLHE41* mRNA (DEC2) | 5’-CGAGACGACACCAAGGAT  ACC-3' | 5’-TCCAGATGTCCCAGAGTTG  TC-3' |
| *DBP* mRNA | 5’-GATCTTGCCCTATCAAGCA  TTCC-3' | 5’-GGCTCCAGTATTTCTCATC  CTTCTG-3' |
| *WEE1* mRNA | 5’-CACACGCCCAAGAGTTT  GC-3' | 5’-AGGAATGAAGCAACAAAG  AATCCG-3' |
| *MAPK11* mRNA | 5’-GCAGGAGCTGAACAAGAC  CG-3' | 5’-TCGTAGGCCGAACAGACG  G-3' |
| *MEX3A* mRNA | 5’-CTGAAGGGCAGCAGCAAC  AC -3' | 5’-GGCCTTAATCTTGCAGCCT  TGC-3' |
| *TEF* mRNA | 5’-ACCGTGTCCAGCACAGAA  TC-3' | 5’-AGTTCACATCCACTTCCAC  AC-3' |
| aKSM eRNA | 5’-TTATGCGAGGTAACCGAG  CG-3’ | 5’-TTTGTCCAACGAGTGCCAG  A-3’ |
| eKSM eRNA | 5’-TTCCTGGTCCCCCAATCTA  GG-3’ | 5’-GCGAGTTCATCTGAGGACA  GT-3’ |
| KDE eRNA | 5’-AGACGATCTCTGGACAGT  ACAC-3’ | 5’-GTCATTTCTGACGTACCGT  GTG-3’ |

**Table S3. CircWave analysis of gene expression**

|  | **MCF10A** | | | | **MDA-MB-231** | | | |
| --- | --- | --- | --- | --- | --- | --- | --- | --- |
| **Gene** | **p-value** | **r2** | **Peak phase [CT; actual]** | **Amplitude**  **(% of mean)** | **p-value** | **r2** | **Peak phase [CT; actual]** | **Amplitude**  **(% of mean)** |
| ***BMAL1* mRNA** | 0 | 0.8452 | 7.432 (31.4) | 149.7% | 2.24 × 10^-5^ | 0.6393 | 11.70 (35.7) | 143.3% |
| ***KLF9* mRNA**  **(pair #1)** | 0.0005 | 0.5118 | 3.313 (27.3) | 100.3% | 0.0422 | 0.2603 | 4.704 (28.7) | 40.53% |
| ***KLF9* mRNA**  **(pair #2)** | 1 × 10^-7^ | 0.7813 | 23.95 | 119.9% |  |  |  |  |
| ***KLF9* pre-mRNA** | 1.89 × 10^-5^ | 0.6450 | 0.1108 (24.1) | 103.2% |  |  |  |  |
| ***PER1* mRNA** | 1.93 × 10^-5^ | 0.6444 | 20.74 | 152.2% | 0.0012 | 0.4732 | 0.0869 (24.1) | 133.2% |
| ***PER2* mRNA** | 0.0003 | 0.5328 | 22.80 | 88.39% |  |  |  |  |
| ***DEC2* mRNA** | 2.2 × 10^-6^ | 0.7107 | 18.59 | 151.5% |  |  |  |  |

Circadian rhythmicity analysis was performed using CircWave (42) for abridged data t = 12 hr to t = 36 hr, as the CircWave software allows for 24-hr period. The α cut-off was set to 0.05 and peak phase is expressed in circadian time (CT; software output) and actual time (e.g. actual 24 hr was expressed as 0 hr when inputted into the program). Amplitude of CircWave curve fit data was calculated as percent of data mean to enable comparison of amplitudes between datasets (43, 44).

**Figure Legends for Additional Files 2 to 14**

**Additional File 2: Figure S1. Baseline *NR3C1* (GR) expression across three breast epithelial lines.** MCF7 expressed considerably lower levels of GR mRNA relative to its triple-negative counterparts, MCF10A and MDA-MB-231 (one-way ANOVA; *P* < 0.0001). Expression levels were normalized the *18s rRNA* housekeeping gene and normalized values were log_10_ transformed prior to statistical analysis. Bars represent mean ± SEM and the letters above the mean indicate significant differences among treatments (means with the same letter are not significantly different, *P* < 0.05, Tukey’s multiple comparison test). All treatments were performed with n=4 replicates and experiments were performed twice with consistent results. Graphs shown are representative of the different trials.

**Additional File 3: Figure S2. Expression of *KLF9* and the direct ER target *GREB1* upon estrogen treatment.** Changes in *KLF9* transcript levels in response to 10 nM E2 treatment from (39) were plotted as TPM over time. **(A)** Induction of *KLF9* by E2 at 30 min abruptly decreased to baseline by 1 hr and continued to decline slowly over time. **(B)** *GREB1* which is directly upregulated by ER signaling served as positive control. **(C)** In MCF7 cells treated with increasing doses of E2, *GREB1* mRNA was induced starting at 10 nM E2 (one-way ANOVA; *P* < 0.0001). Expression levels in the dose-response experiment were normalized to the *GAPDH* gene, and normalized values were log_10_ transformed before statistical analysis. Bars represent mean ± SEM with letters above the mean indicating significant differences among treatments (means with the same letter are not significantly different; *P* < 0.05, Tukey’s multiple comparisons test). All treatments were performed with 3-4 replicates and all experiments were performed twice with consistent results.

**Additional File 4: Figure S3. GR localization in the *KLF9* proximal promoter along with the eKSM and aKSM.** The UCSC genome browser (50) was used to visualize the *KLF9* locus and surrounding non-coding regions mapped to the human GRCh37/hg19 genome assembly. Highlighted are the proximal promoter (3kb upstream of TSS; green), eKSM (blue), and aKSM (yellow). In contrast to the eKSM and aKSM which are enriched for GR localization in both MCF10A (45) and MDA-MB-231 (46) cells, as well as RNA Pol II binding (45), active enhancer marks as demonstrated by analysis of ENCODE data for non-coding transcripts based on RNA-seq, H3K27Ac ChIP-seq peaks, DNase I Hypersensitivity Clusters (47), and predicted long-range interaction with the KLF9 promoter from the GeneHancer database (49), the proximal promoter is not enriched for these active chromatin marks and have minimal to no GR and RNA pol II binding.

**Figure S4. Transcription factor response elements in the eKSM, aKSM, and KDE.** In each of the enhancers, LASAGNA search 2.0 (51) was used to identify GREs and EREs, while CLOCK-binding sites were manually determined based on previously derived sequences in order of decreasing induction by CLOCK in a reporter enhancer assay as previously described (EboxA = CACGTG, EboxB = CACGTT or AACGTG EboxC = CACGCG, EboxE = CACGAG) (15).

**Additional File 6: Figure S5. CORT-dependent transcription of enhancer RNAs (eRNA) from the aKSM, eKSM, and KDE is a direct effect of GR activity.** MCF10A cells were pre-incubated with 100 μg/mL CHX for 30 min or 1 μM MIF before treatment with 300 nM CORT for 2 hr. **(A-F)** Nascent enhancer RNA transcription at basal conditions can be detected from all three *KLF9* enhancer regions: eKSM, aKSM, and KDE. **(A, C, E)** CORT treatment significantly induced eRNA transcription at all three regions that is not altered in the presence of the protein synthesis inhibitor CHX (Student’s *t*-test; *P* < 0.001). **(B, D, E)** CORT-dependent transcription of the eRNAs is GR-specific as pre-incubation with the GR-selective antagonist MIF abolished the increase in eRNA transcript (one-way ANOVA; eKSM: *P* < 0.0001; aKSM: *P* = 0.0007; KDE: *P* = 0.0012). Enhancer RNA expression was normalized to the *18s rRNA* reference gene which was unaffected by hormone treatment, and normalized values were log_10_ transformed before statistical analysis. Bars represent mean ± standard error of the mean with statistically significant difference indicated by asterisks in Student’s *t-*test (**P* <0.001, ***P* < 0.0001) or letters above the means in one-way ANOVA (means with the same letter are not significantly different; *P* < 0.05, Tukey’s multiple comparisons test). All treatments were performed with 3-4 replicates and all experiments were performed twice with consistent results.

**Additional File 7: Figure S6. *KLF9* transcript time-course expression in human cell lines and *in vivo* murine mammary epithelia*.* (A-D)** MCF10A cells were pulsed with 1 μM CORT for 2 hr to synchronize circadian gene expression prior to collection of RNA every 4 hr. Circadian expression of both *KLF9* **(A, B)** mRNA and **(C, D)** pre-mRNA (blue lines) was antiphase with the expression of *BMAL1* (black) both peaking at 24 hr concurrent with *BMAL1* expression nadir. **(A, B)** Expression of *KLF9* mRNA, assayed through another primer set in RT-qPCR, was still rhythmic with period determined to be 25.68 hr. **(C, D)** This is consistent with the oscillation of *KLF9* pre-mRNA with period calculated to be 26.47 hr. (**E, F**) Transcriptome analysis of the mouse breast circadian clock was performed by obtaining time-series microarray data from Yang et al. (54) where mammary tissues were isolated every 4 hr for 48 hr from mice kept under total darkness. Processed expression data was downloaded from the Array Express database (E-MTAB-5330) and **(E)** fold change in one *Klf9* probeset (1428289_at) was calculated based on the earliest time point, demonstrating that *Klf9* expression mirrors a circadian pattern, peaking at circadian time 11 (CT11) and CT35. **(F)** To further provide evidentiary support, original *Klf9* probe data was linearly detrended and fitted to a cosine wave model with 24-hr periodicity, with period calculated to be 26.14 hr. This is in agreement with the findings of Yang and colleagues (54) wherein *Klf9* was listed as one of the genes that were expressed in a circadian pattern in the mouse mammary gland (24-cosine-wave model, CircWave Batch version 5). Expression levels were normalized to the *GAPDH* housekeeping genes and normalized values were log_10_ transformed prior to statistical analysis. Dashed lines indicate confidence bands demarcating the likely location of the true curve at 95% confidence level. All treatments were performed with 4 replicates and experiments were performed twice with consistent results.

**Additional File 8: Figure S7. Circadian expression of *PER1, PER2,* and *DEC2* mRNA in MCF10A cells and *PER1* in MDA-MB-231 cells. (A-F)** MCF10A and **(G-H)** MDA-MB-231 cells were pulsed with 1 μM CORT for 2 hr to synchronize circadian gene expression prior to collection of RNA every 4 hr. Circadian expression of BMAL1 target genes (blue) **(A, B)** *PER1,* **(C, D)** *PER2,* and **(E, F)** *DEC2* was antiphase with the expression of *BMAL1* (black), with maximal target transcript levels occurring at *BMAL1* nadir (t = 24 hr). (**B, D, F**) All *BMAL1* targets have periods around 24 hr as determined through cosine-wave regression analyses. **(G)** The antiphase oscillation of *BMAL1* and *PER1* is conserved in the MDA-MB-231 line. However, rhythmic oscillation of both genes is slightly aberrant, with *BMAL1* expression plateauing at t = 36 hr and **(H)** *PER1* abruptly increasing at t = 48 hr in contrast to what is observed in MCF10A. Expression levels were normalized to the *GAPDH* housekeeping genes and normalized values were log_10_ transformed prior to statistical analysis. Dashed lines indicate confidence bands demarcating the likely location of the true curve at 95% confidence level. All treatments were performed with 4 replicates and experiments were performed twice with consistent results.

**Additional File 9: Figure S8. Overlap in KLF9 and CLOCK binding to genomic loci of core clock and clock target genes.** Publicly available KLF9 (GSE105301) and CLOCK (GSE127640) ChIP-seq data in MCF7 cells (47) were obtained from Gene Expression Omnibus and visualized using the UCSC genome browser (50) . Clock gene loci and surrounding non-coding regions were mapped to the human February 2009 (GRCh37/hg19) genome assembly. In black-outlined boxes are KLF9 peaks which almost always co-localize with CLOCK in the same cell line.

**Additional File 10: Figure S9. Validation of *KLF9* knockdown and overexpression in** **mammary epithelial cell models.** **(A, C, E)** Human *KLF9* was knocked down in MCF10A, MCF7, and MDA-MB-231 via lentiviral-mediated transduction of one of two *KLF9* shRNAs (shKLF9-3: TRCN0000013630; shKLF9-4: TRCN0000013631). *KLF9* expression was consistently reduced between scrambled and knockdown cells in each cell line for shKLF9-3 (Student’s *t-*test; MCF10A: *P* < 0.0001; MCF7: *P* = 0.0001; MDA-MB-231: *P* < 0.0001), but not shKLF9-4 (Student’s *t-*test; MCF10A: *P* = 0.0419; MCF7: *P =* 0.7385; MDA-MB-231: *P* = 0.0077). **(B, D, F)** *KLF9* was successfully overexpressed in all three epithelial cell models (Student’s *t-*test; MCF10A: *P* < 0.0001; MCF7: *P* < 0.0001; MDA-MB-231: *P* < 0.0001). Percentage knockdown or overexpression of *KLF9* mRNA in each shRNA/construct relative to the control is indicated above the mean. *KLF9* expression was normalized to the *18s rRNA* reference gene and normalized values were log_10_ transformed before statistical analysis. Experiments were performed with n = 4 replicates and bars represent mean ± SEM with statistical significance relative to scrambled or empty vector control indicated by hashes in Student’s *t*-test (^####^*P* < 0.0001).

**Additional File 11: Figure S10. Validation of KLF9 targets upon *KLF9* knockdown or overexpression in three breast epithelial cell lines.** Effects of *KLF9* **(A, C, E)** knockdown and **(B, D, E)** overexpression on the expression of established KLF9*-*repressed targets *DBP* (16)*, MAPK11* (55)*,* and *MEX3A* (55) in **(A, C)** MCF10A, **(B, D)** MCF7, and **(E, F)** MDA-MB-231 cells as measured through RT-qPCR. In MCF10A, **(A)** knockdown of *KLF9* increased the expression of *MAPK11* and *MEX3A* mRNA (Student’s *t-*test; *MAPK11: P =* 0.0155, *MEX3A: P =* 0.0082), **(B)** whereas overexpression conferred the converse effect of downregulating the expression of *DBP, MAPK11,* and *MEX3A* transcript levels (Student’s *t-*test; *DBP: P =* 0.0047, *MAPK11: P =* 0.0261, *MEX3A: P =* 0.0054). **(C)** Knockdown of *KLF9* in MCF7 cells led to a significant increase in *DBP* expression, as well a trend in upregulation of *MAPK11* mRNA (Student’s *t-*test; *DBP: P =* 0.0009, *MAPK11: P =* 0.1104). **(D)** This is consistent with the observed decrease in *MAPK11* and trend of downregulation in *DBP* transcript levels upon *KLF9* overexpression (Student’s *t-*test; *DBP: P =* 0.0927, *MAPK11: P =* 0.0349). **(E)** These were generally consistent in MDA-MB-231 cells, with *KLF9* knockdown resulting in increased *MAPK11* mRNA levels and a trend of upregulation in *DBP* transcript (Student’s *t-*test; *DBP: P =* 0.0788, *MAPK11: P =* 0.0001), while overexpression concordantly downregulated all three *KLF9*-downregulated targets (Student’s *t-*test; *DBP: P =* 0.0196, *MAPK11: P =* 0.0001, *MEX3A: P =* 0.0041). Expression was normalized to the *18S rRNA* reference gene which was unaffected by genetic perturbation, and normalized values were log_10_ transformed before statistical analysis. Bars represent mean ± standard error of the mean with statistically significant difference indicated by hashes in Student’s *t-*test (^#^*P* < 0.05, ^##^*P* < 0.01, ^###^*P* < 0.001). All treatments were performed with 4 replicates and all experiments were performed twice with consistent results.

**Additional File 12: Figure S11. Genetic perturbation of *KLF9* moderately influences expression of other clock genes.** Effects of *KLF9* **A, B)** knockdown and **C, D** overexpression on the expression of core clock and clock output genes in **A, C** MCF10A and **B, D** MDA-MB-231 cells as measured through RT-qPCR. *KLF9* knockdown resulted in a significant increase in expression of *CRY2, NR1D2* (REV-ERBβ)*,* and *DEC1* in MCF10A cells (Student’s *t* -test; CRY2: *P =* 0.0225, NR1D2: *P =* 0.0322, DEC1: *P* = 0.0012). **(B)** The increase is consistent for *CRY2* in MDA-MB-231 *KLF9* knockdown cells (Student’s *t*-test; *CRY2*: *P* = 0.0136). **C)** On the other hand, overexpression of *KLF9* in MCF10A led to an increase in *CRY1, NR1D1, NR1D2* (Student’s *t-*test; *CRY1*: *P =* 0.0374, *NR1D1*: *P =* 0.0013, *NR1D2*: *P =* 0.0363). **D** This was mostly consistent in MDA-MB-231 cells except for an observed increase in *CRY2* and *TEF* and a decrease in *DEC1* transcript levels (Student’s *t*-test; *CRY2*: *P =* 0.0005, *NR1D1*: *P* = 0.0056, *DEC1*: *P =* 0.0091, *TEF*: *P =* 0.0215). Expression was normalized to the *18S rRNA* reference gene which was unaffected by genetic perturbation, and normalized values were log_10_ transformed before statistical analysis. Bars represent mean ± standard error of the mean with statistically significant difference indicated by hashes in Student’s *t-*test (^#^*P* < 0.05, ^##^*P* < 0.01, ^###^*P* < 0.001, ^####^*P* < 0.0001). All treatments were performed with 4 replicates and all experiments were performed twice with consistent results.

**Additional File 13: Figure S12. Overexpression of *KLF9* abrogates E2 induction of *GREB1* in ER+ MCF7 cells.** MCF7 cells were treated with 1 μM E2 for 24 hr prior to analysis of gene expression. *GREB1* mRNA was induced upon E2 treatment in empty vector control MCF7 cells while *KLF9* overexpression attenuated the induction (two-way ANOVA; Treatment: *P* < 0.0001; Overexpression: *P* = 0.0020; Interaction: *P* = 0.0057). Expression levels were normalized to the *18S rRNA* housekeeping gene which was unaffected by hormone treatment, and normalized values were log_10_ transformed before statistical analysis. Bars represent mean ± SEM with statistical significance determined through Student’s *t*-test (*P* < 0.0001 for statistically significant effect of E2 within an expression vector type, and ^#^*P* < 0.001for statistically significant effects of KLF9 overexpression between the same treatment).

**Additional File 14: Figure S13. Effects of *KLF9* knockdown or overexpression and CORT treatment on colony formation and viability of breast epithelial cells.** Cell survival **(A-F)** and viability **(G-L)** were assessed in *KLF9* **(A, C, E, G, I, K)** -knockdown and **(B, D, F, H, J, L)** -overexpressing cells treated with either vehicle or CORT (100 nM) using the colony formation and resazurin reduction assays, respectively. For the colony formation assay, representative images of colonies of **(A, B)** MCF10A, **(C, D)** MCF7, and **(E, F)** MDA-MB-231 cells stained with crystal violet after 14-day treatment. For the cell viability assay, CORT treatment promoted cell proliferation in **(G, H)** MCF10A and **(K, L)** MDA-MB-231 cells, but had opposite effects in **(I, J)** MCF7 cells. CORT enhanced colony formation and viability in triple-negative MCF10A and MDA-MB-231 while it conferred the converse anti-tumorigenic effects in the ER+ MCF7 line. Moreover, *KLF9* generally restricts cell survival and proliferation in all three breast epithelial lines. Viability measurements were normalized to raw fluorescence reads at 0 hr as the baseline, and derived normalized values were log_10_ transformed before statistical analysis. Lines represent mean fold induction ± SEM. Treatments were done with 4-5 replicates and experiments were performed twice with consistent results.
