# Supplementary figures and images for: Krüppel-like factor 9 (KLF9) links hormone dysregulation and circadian disruption to breast cancer pathogenesis
Source: Cancer Cell Int. 2023 Feb 23;23:33. doi: 10.1186/s12935-023-02874-1 (PMC9948451; doi:10.1186/s12935-023-02874-1)

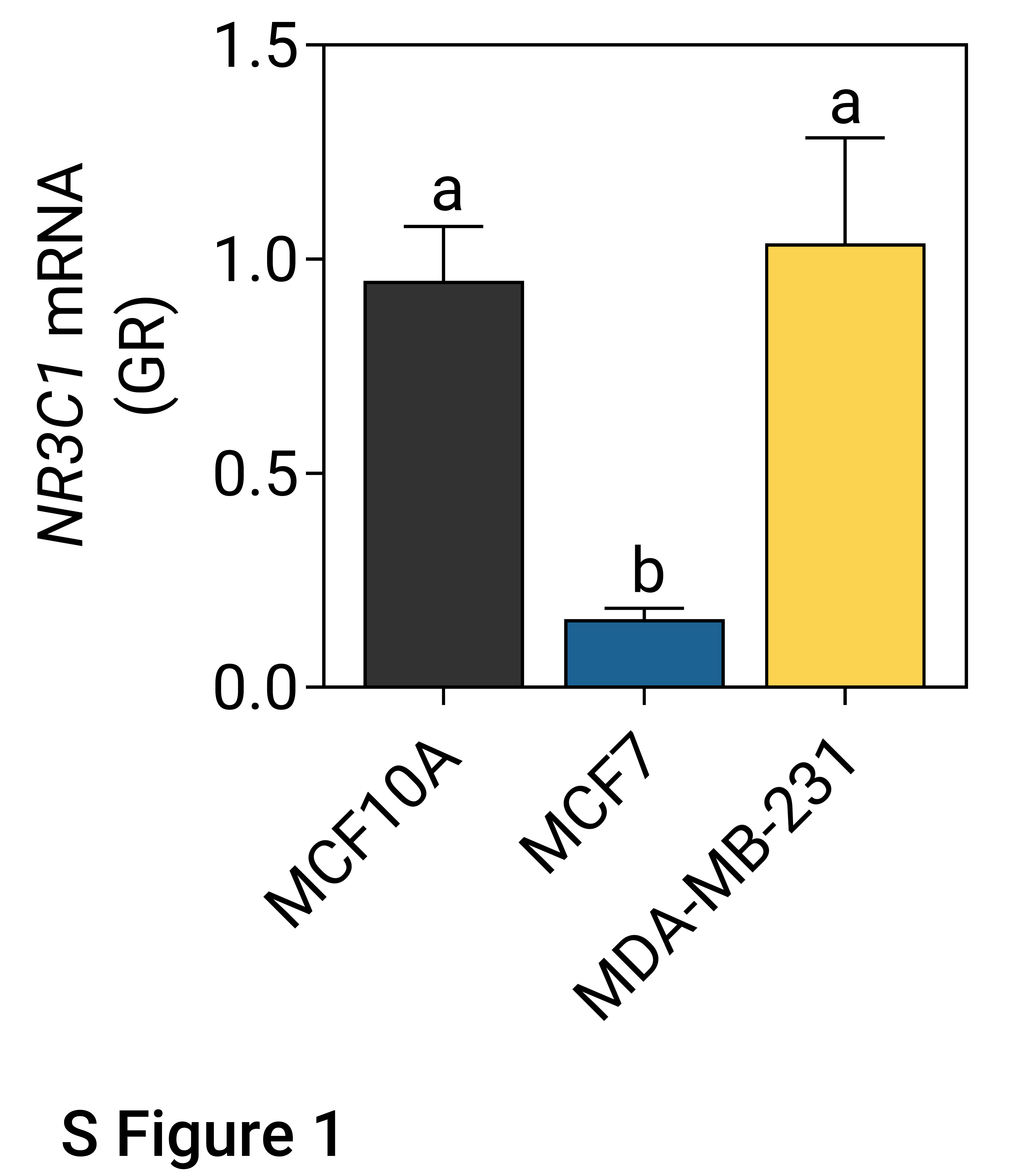

Supplement: Supplementary file 2 — Additional file 2: Figure S1. Baseline NR3C1 (GR) expression across three breast epithelial lines. MCF7 expressed considerably lower levels of GR mRNA relative to its triple-negative counterparts, MCF10A and MDA-MB-231 (one-way ANOVA; P < 0.0001). [file 12935_2023_2874_MOESM2_ESM.tif]

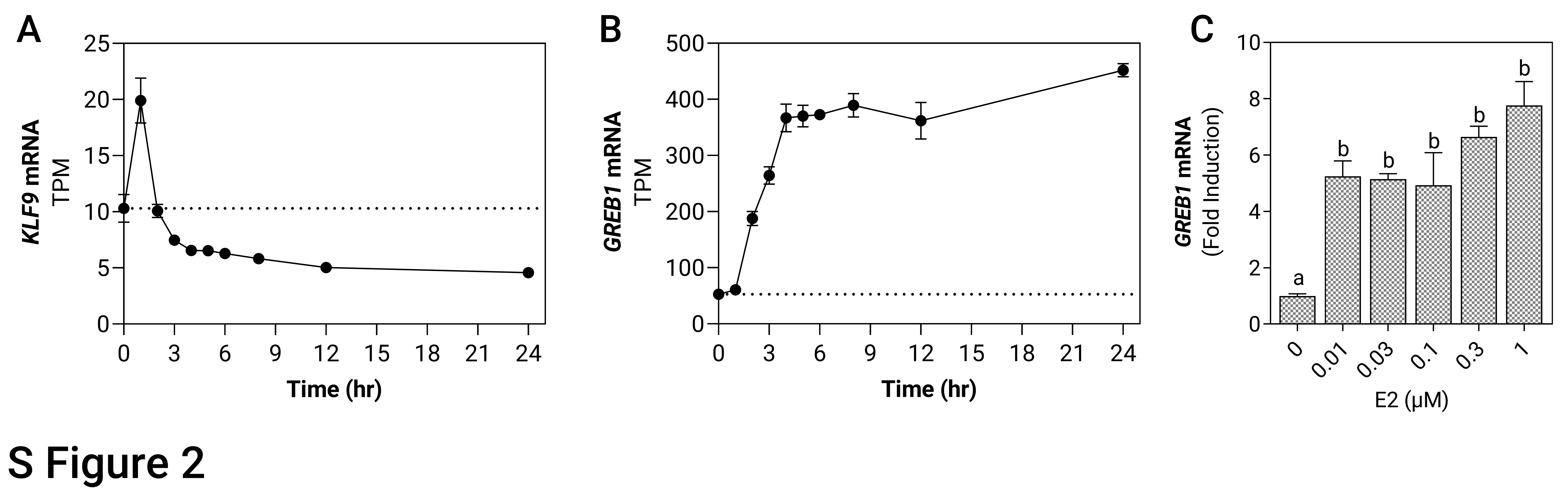

Supplement: Supplementary file 3 — Additional file 3: Figure S2. Expression of KLF9 and the direct ER target GREB1 upon estrogen treatment. Changes in KLF9 transcript levels in response to 10 nM E2 treatment from (39) were plotted as TPM over time. (A) Induction of KLF9 by E2 at 30 min abruptly decreased to baseline by 1 hr and continued to decline slowly over time. (B) GREB1 which is directly upregulated by ER signaling served as positive control. (C) In MCF7 cells treated with increasing doses of E2, GREB1 mRNA was induced starting at 10 nM E2 (one-way ANOVA; P < 0.0001). [file 12935_2023_2874_MOESM3_ESM.tif]

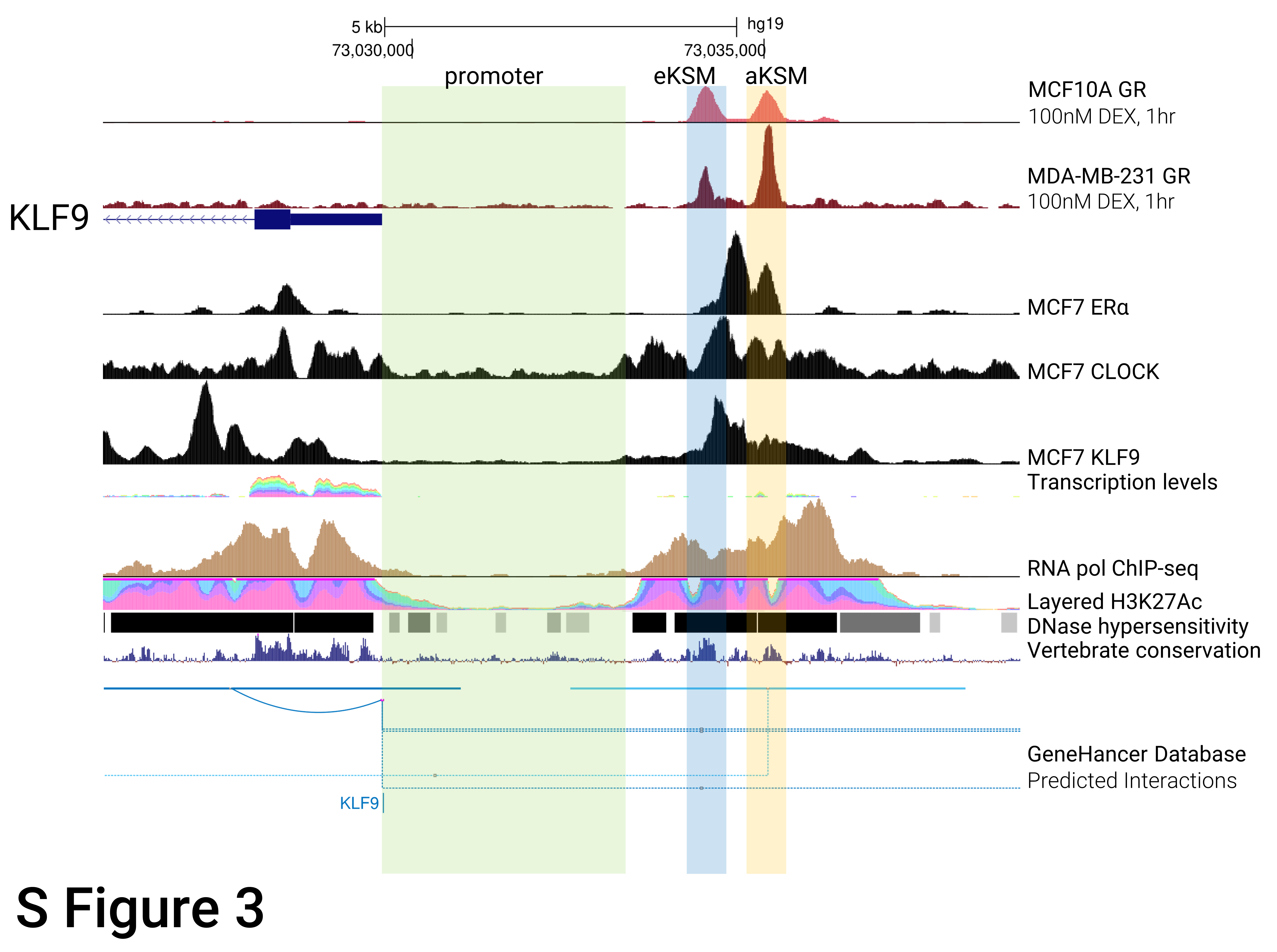

Supplement: Supplementary file 4 — Additional file 4: Figure S3. GR localization in the KLF9 proximal promoter, eKSM and aKSM. The UCSC genome browser (47) was used to visualize the KLF9 locus and surrounding non-coding regions mapped to the human GRCh37/hg19 genome assembly. Highlighted are the proximal promoter (3kb upstream of TSS; green), eKSM (blue), and aKSM (yellow). [file 12935_2023_2874_MOESM4_ESM.tif]

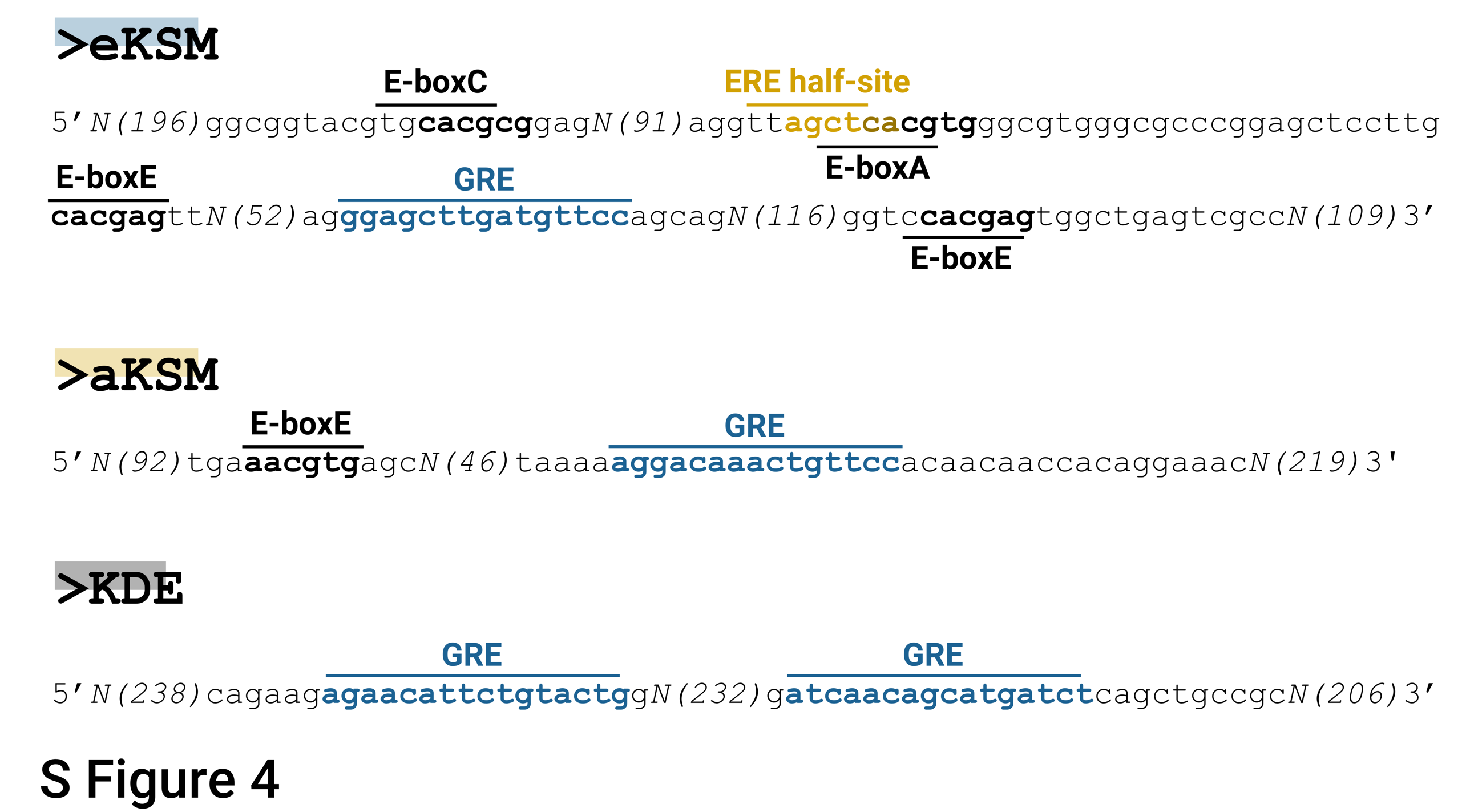

Supplement: Supplementary file 5 — Additional file 5: Figure S4. Transcription factor response elements in the eKSM, aKSM, and KDE. In each of the enhancers, LASAGNA search 2.0 (48) was used to identify GREs and EREs, while CLOCK-binding sites were manually determined based on previously derived sequences in order of decreasing induction by CLOCK in a reporter enhancer assay as previously described (EboxA = CACGTG, EboxB = CACGTT or AACGTG EboxC = CACGCG, EboxE = CACGAG) (15). [file 12935_2023_2874_MOESM5_ESM.tif]

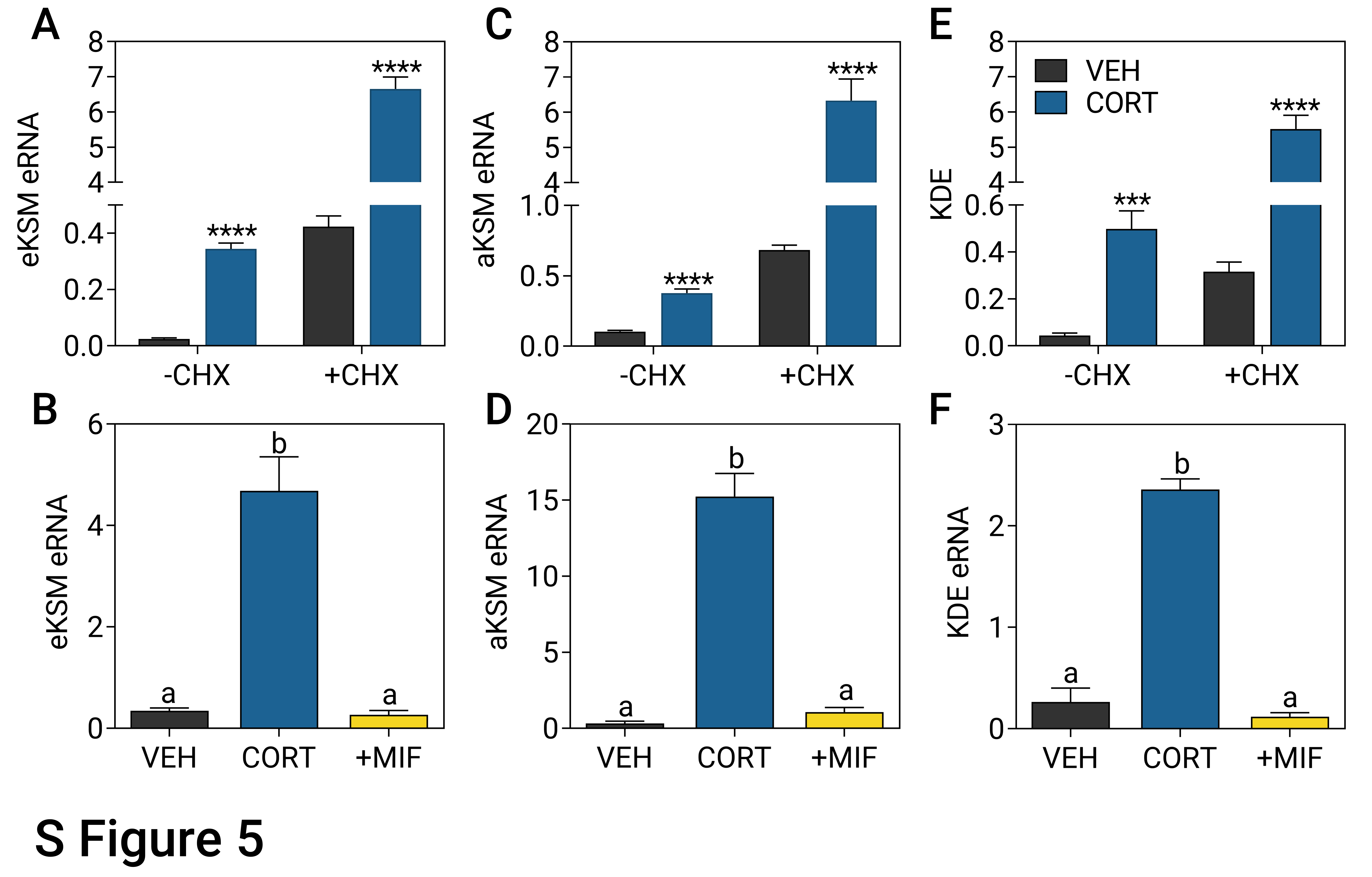

Supplement: Supplementary file 6 — Additional file 6: Figure S5. CORT-dependent transcription of enhancer RNAs (eRNA) from the aKSM, eKSM, and KDE is a direct effect of GR activity. MCF10A cells were pre-incubated with 100 μg/mL CHX for 30 min or 1 μM MIF before treatment with 300 nM CORT for 2 hr. (A-F) Nascent enhancer RNA transcription at basal conditions can be detected from all three KLF9 enhancer regions: eKSM, aKSM, and KDE. (A, C, E) CORT treatment significantly induced eRNA transcription at all three regions that is not altered in the presence of the protein synthesis inhibitor CHX (Student’s t-test; P < 0.001). (B, D, E) CORT-dependent transcription of the eRNAs is GR-specific as pre-incubation with the GR-selective antagonist MIF abolished the increase in eRNA transcript (one-way ANOVA; eKSM: P < 0.0001; aKSM: P = 0.0007; KDE: P = 0.0012). [file 12935_2023_2874_MOESM6_ESM.tif]

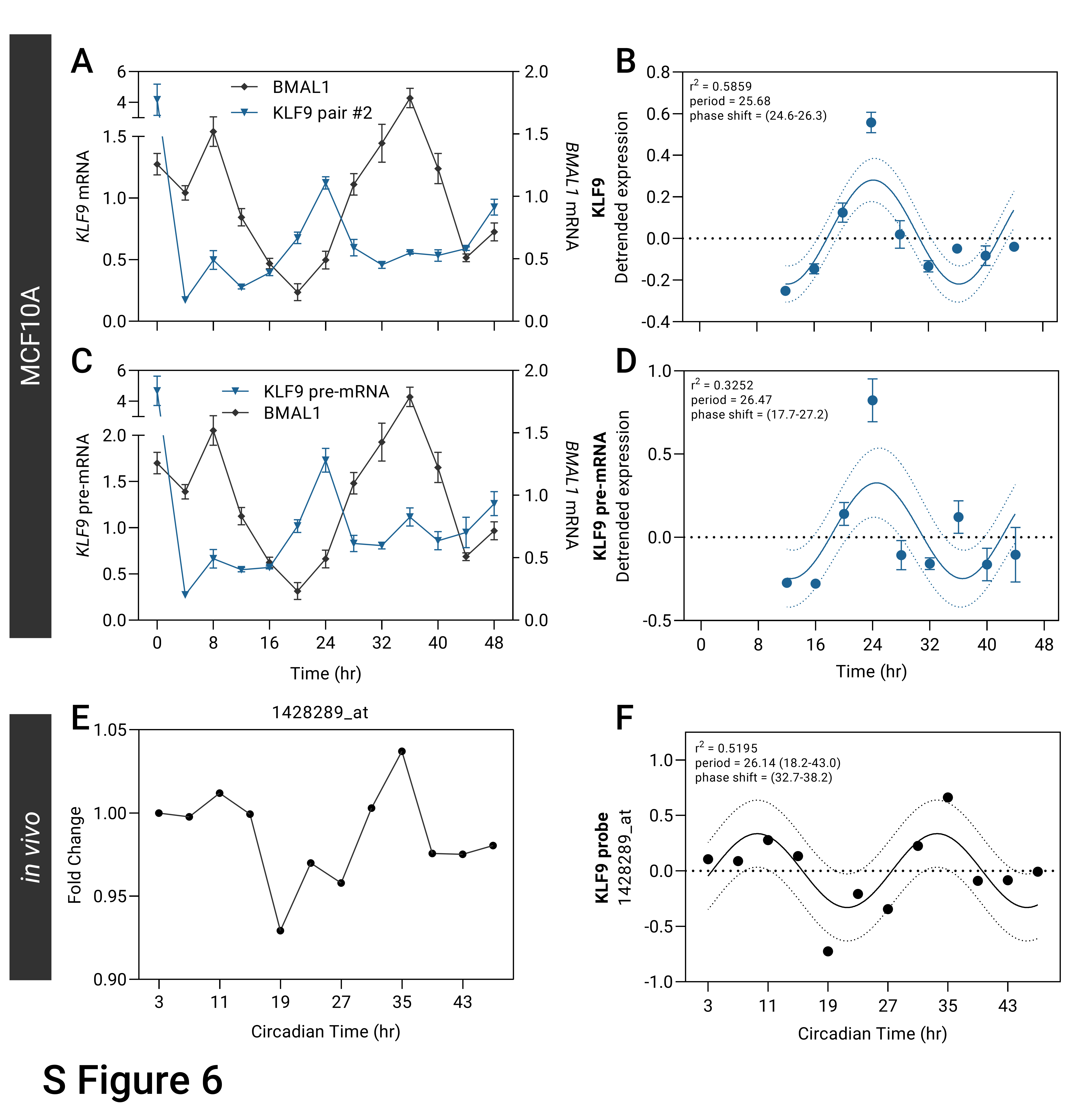

Supplement: Supplementary file 7 — Additional file 7: Figure S6. KLF9 transcript time-course expression in human cell lines and in vivo murine mammary epithelia. (A-D) MCF10A cells were pulsed with 1 μM CORT for 2 hr to synchronize circadian gene expression prior to collection of RNA every 4 hr. Circadian expression of both KLF9 (A, B) mRNA and (C, D) pre-mRNA (blue lines) was antiphase with the expression of BMAL1 (black) both peaking at 24 hr concurrent with BMAL1 expression nadir. (A, B) Expression of KLF9 mRNA, assayed through another primer set in RT-qPCR, was still rhythmic with period determined to be 25.68 hr. (C, D) This is consistent with the oscillation of KLF9 pre-mRNA with period calculated to be 26.47 hr. (E, F) Transcriptome analysis of the mouse breast circadian clock was performed by obtaining time-series microarray data from Yang et al. (54) where mammary tissues were isolated every 4 hr for 48 hr from mice kept under total darkness. [file 12935_2023_2874_MOESM7_ESM.tif]

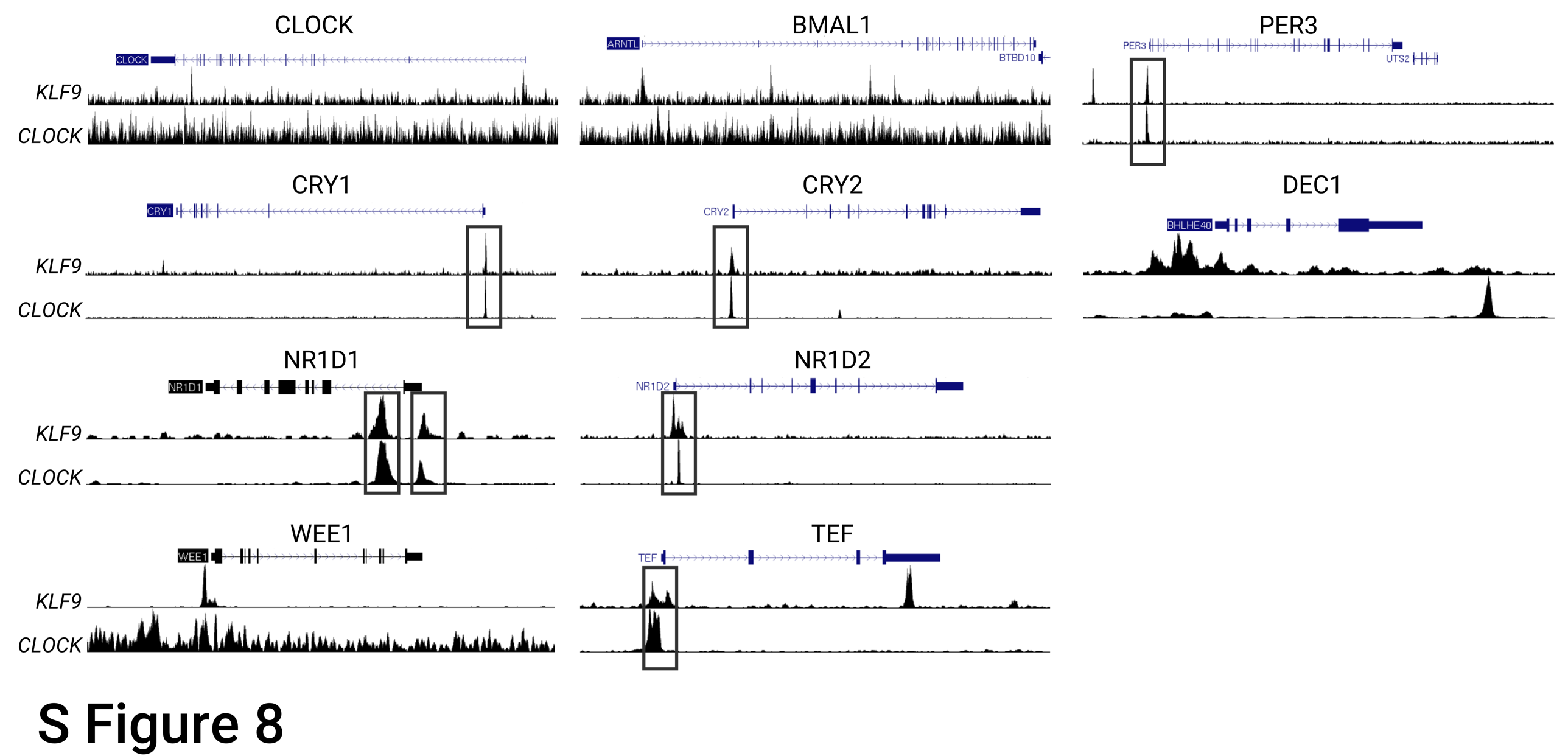

Supplement: Supplementary file 9 — Additional file 9: Figure S8. Overlap in KLF9 and CLOCK binding to genomic loci of core clock and clock target genes. Publicly available KLF9 (GSE105301) and CLOCK (GSE127640) ChIP-seq data in MCF7 cells (47) were obtained from Gene Expression Omnibus and visualized using the UCSC genome browser (50) . Clock gene loci and surrounding non-coding regions were mapped to the human February 2009 (GRCh37/hg19) genome assembly. In black-outlined boxes are KLF9 peaks which almost always co-localize with CLOCK in the same cell line. [file 12935_2023_2874_MOESM9_ESM.tif]

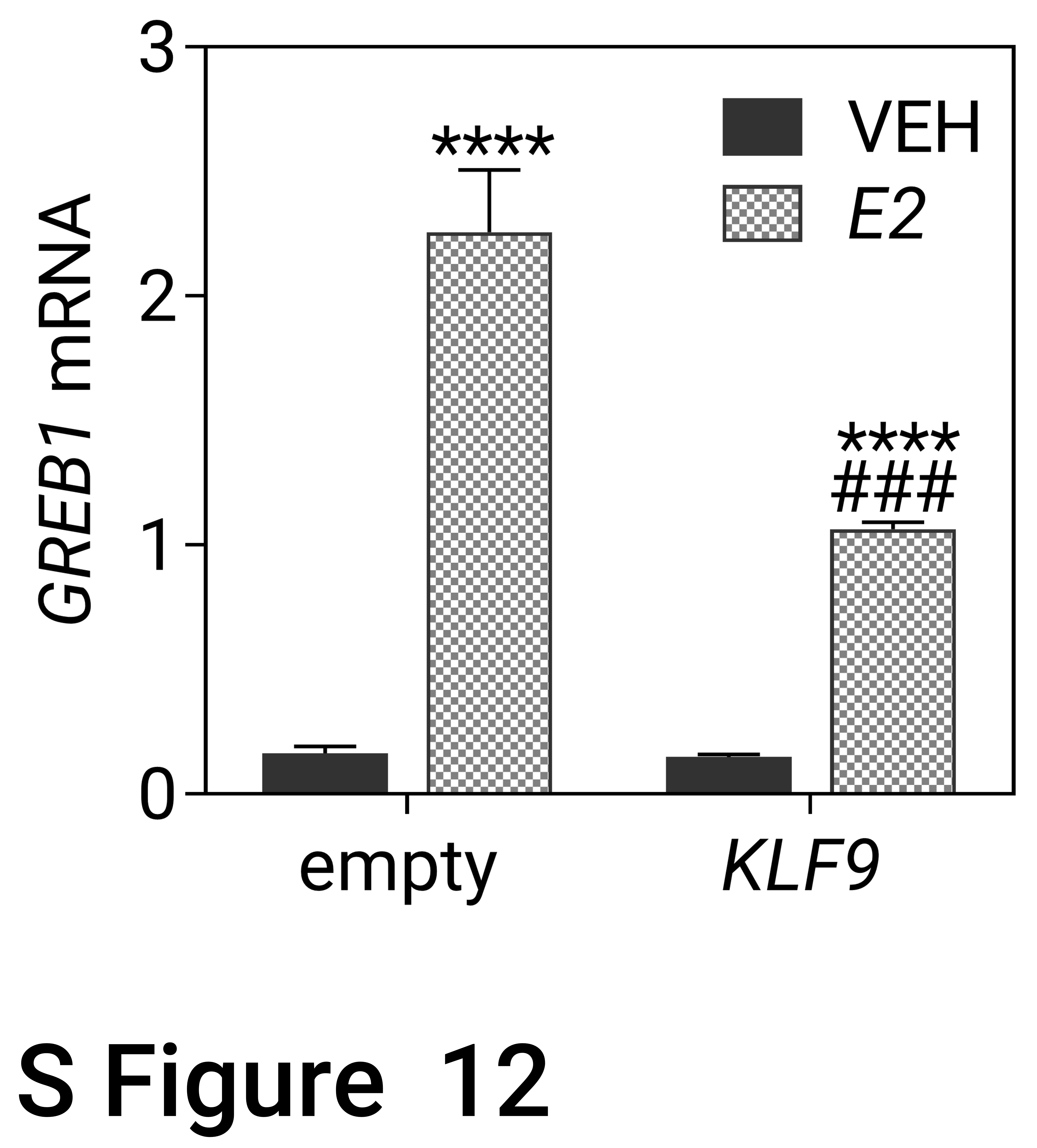

Supplement: Supplementary file 13 — Additional file 13: Figure S12. Overexpression of KLF9 abrogates E2 induction of GREB1 in ER+ MCF7 cells. MCF7 cells were treated with 1 μM E2 for 24 hr prior to analysis of gene expression. GREB1 mRNA was induced upon E2 treatment in empty vector control MCF7 cells while KLF9 overexpression attenuated the induction (two-way ANOVA; Treatment: P < 0.0001; Overexpression: P = 0.0020; Interaction: P = 0.0057). [file 12935_2023_2874_MOESM13_ESM.tif]

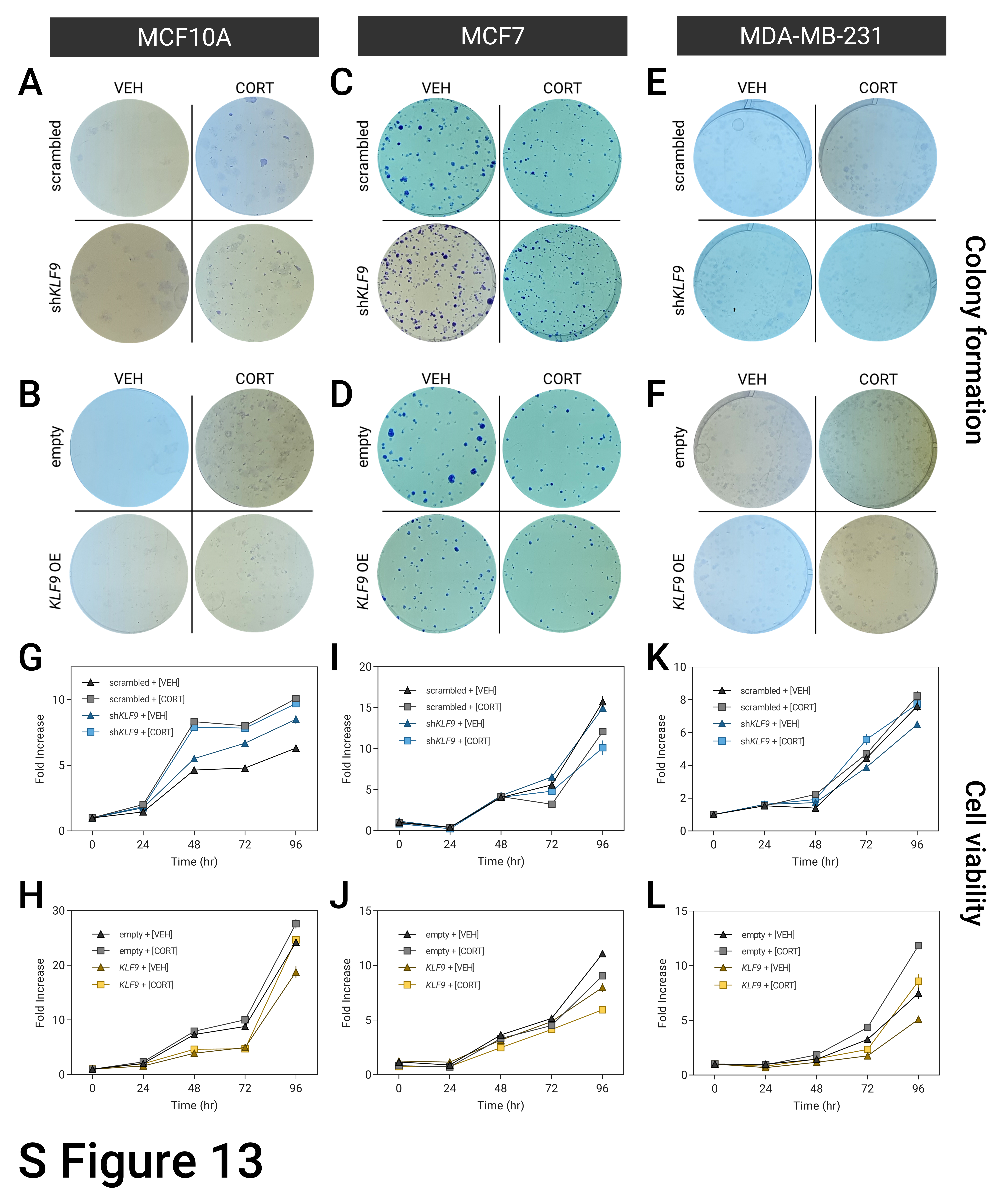

Supplement: Supplementary file 14 — Additional file 14: Figure S13. Effects of KLF9 knockdown or overexpression and CORT treatment on colony formation and viability of breast epithelial cells. Cell survival (A-F) and viability (G-L) were assessed in KLF9 (A, C, E, G, I, K) -knockdown and (B, D, F, H, J, L) -overexpressing cells treated with either vehicle or CORT (100 nM) using the colony formation and resazurin reduction assays, respectively. For the colony formation assay, representative images of colonies of (A, B) MCF10A, (C, D) MCF7, and (E, F) MDA-MB-231 cells stained with crystal violet after 14-day treatment. For the cell viability assay, CORT treatment promoted cell proliferation in (G, H) MCF10A and (K, L) MDA-MB-231 cells, but had opposite effects in (I, J) MCF7 cells. [file 12935_2023_2874_MOESM14_ESM.tif]
